# Convergent evolution of tRNA gene targeting preferences in compact genomes

**DOI:** 10.1186/s13100-016-0073-9

**Published:** 2016-08-31

**Authors:** Thomas Spaller, Eva Kling, Gernot Glöckner, Falk Hillmann, Thomas Winckler

**Affiliations:** 1 Institute of Pharmacy, Department of Pharmaceutical Biology, Friedrich Schiller University Jena, Semmelweisstraße 10, Jena, 07743 Germany; 2 Institute for Biochemistry I, Medical Faculty, University of Cologne, Berlin, Germany; 3 Institute for Freshwater Ecology and Inland Fisheries, IGB, Berlin, Germany; 4 Junior Research Group Evolution of Microbial Interaction, Leibniz Institute for Natural Product Research and Infection Biology—Hans Knöll Institute, Jena, Germany

**Keywords:** *Dictyostelium*, Chromo domain, Chromovirus, Ty3, RNA polymerase III

## Abstract

**Background:**

In gene-dense genomes, mobile elements are confronted with highly selective pressure to amplify without causing excessive damage to the host. The targeting of tRNA genes as potentially safe integration sites has been developed by retrotransposons in various organisms such as the social amoeba *Dictyostelium discoideum* and the yeast *Saccharomyces cerevisiae*. In *D. discoideum*, tRNA gene-targeting retrotransposons have expanded to approximately 3 % of the genome. Recently obtained genome sequences of species representing the evolutionary history of social amoebae enabled us to determine whether the targeting of tRNA genes is a generally successful strategy for mobile elements to colonize compact genomes.

**Results:**

During the evolution of dictyostelids, different retrotransposon types independently developed the targeting of tRNA genes at least six times. DGLT-A elements are long terminal repeat (LTR) retrotransposons that display integration preferences ~15 bp upstream of tRNA gene-coding regions reminiscent of the yeast Ty3 element. Skipper elements are chromoviruses that have developed two subgroups: one has canonical chromo domains that may favor integration in centromeric regions, whereas the other has diverged chromo domains and is found ~100 bp downstream of tRNA genes. The integration of *D. discoideum* non-LTR retrotransposons ~50 bp upstream (TRE5 elements) and ~100 bp downstream (TRE3 elements) of tRNA genes, respectively, likely emerged at the root of dictyostelid evolution. We identified two novel non-LTR retrotransposons unrelated to TREs: one with a TRE5-like integration behavior and the other with preference ~4 bp upstream of tRNA genes.

**Conclusions:**

Dictyostelid retrotransposons demonstrate convergent evolution of tRNA gene targeting as a probable means to colonize the compact genomes of their hosts without being excessively mutagenic. However, high copy numbers of tRNA gene-associated retrotransposons, such as those observed in *D. discoideum*, are an exception, suggesting that the targeting of tRNA genes does not necessarily favor the amplification of position-specific integrating elements to high copy numbers under the repressive conditions that prevail in most host cells.

**Electronic supplementary material:**

The online version of this article (doi:10.1186/s13100-016-0073-9) contains supplementary material, which is available to authorized users.

## Background

Mobile elements are obligate genomic parasites that amplify as selfish DNA and play important roles in driving the evolution of their hosts [1–5]. Retrotransposons mobilize by reverse transcription of RNA intermediates and integration of the resulting DNA copies at new locations of their host’s genomes. Retrotransposons encode proteins that mediate their mobility and they can be distinguished by their overall structures and retrotransposition mechanisms [6]. The supergroup of retrotransposons bearing long terminal repeats (LTRs) is classified into vertebrate retroviruses (Retroviridae), hepadnaviruses, caulimoviruses, Ty1/*copia* (Pseudoviridae), Ty3/*gypsy* (Metaviridae), BEL, and DIRS (*Dictyostelium* intermediate repeat sequence) [7–9]. Non-LTR retrotransposons are a diverse group of mobile elements that lack LTRs and can be further distingushied by structural features such as the presence of an encoded apurinic or apyrimidinic site DNA repair endonuclease or a type IIS restriction endonuclease instead of a retroviral integrase and the presence or absence of a ribonuclease H (RNH) domain as part of the reverse transcriptase (RT) [10, 11].

Dictyostelids are soil-dwelling protists that belong to the supergroup of Amoebozoa [12, 13]. Unfavorable environmental conditions, such as a lack of food, triggers social behaviors in single cells that aggregate and form fruiting bodies to spread some of the population as dormant spores into the environment [14, 15]. *Dictyostelium discoideum*, the model organism in studying the biology of social amoebae, has a 34-Mb haploid genome in which two thirds of the chromosomal DNA code for proteins and intergenic regions are mostly below 1 kb in length [16]. The gene density of this genome limits the available space for transposable elements to expand without causing damage to the host. Therefore, it is remarkable that the genome of *D. discoideum* is interspersed with a variety of mobile elements that add up to nearly 10 % of nuclear DNA [17].

**Fig. 1:**
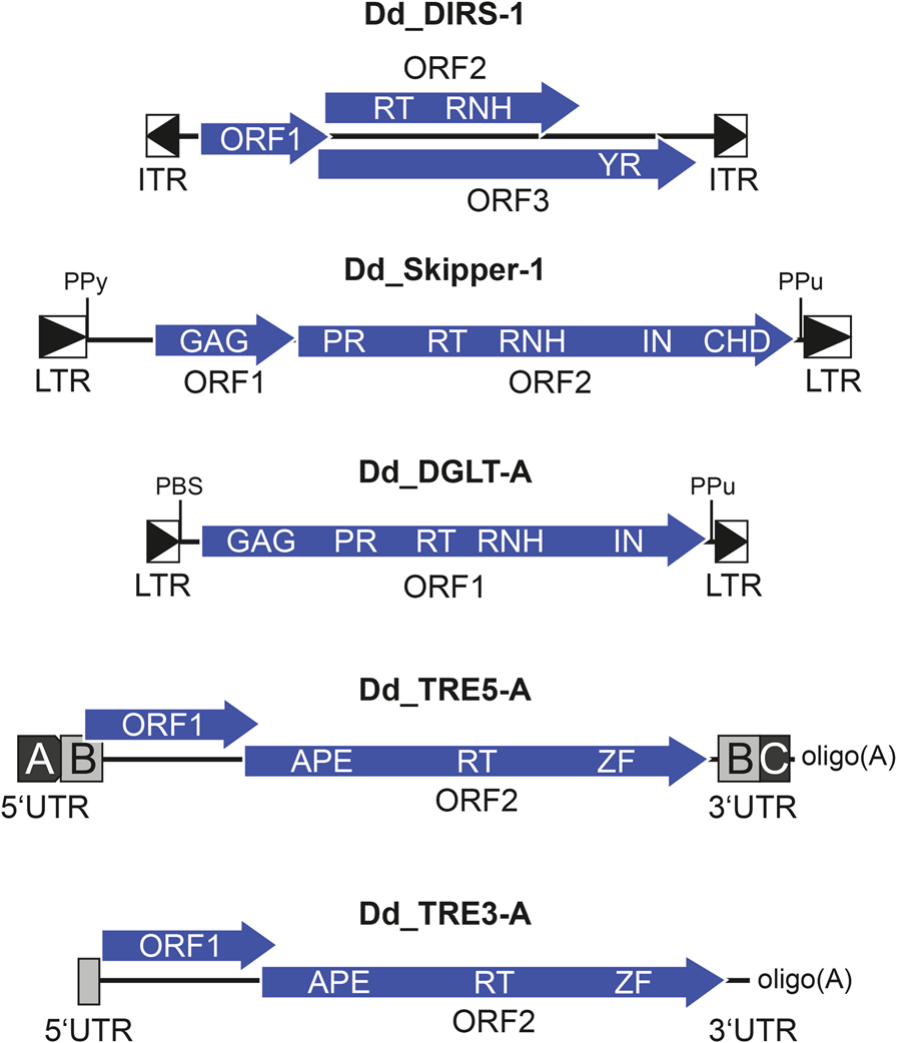
Overview of retrotransposons in the *D. discoideum* genome. DIRS-1 is the founding member of the class of tyrosine recombinase retrotransposons. DIRS-1 contains inverted terminal repeats (ITRs) and three ORFs. ORF1 codes for a protein of unknown function. ORF2 overlaps with ORF3 in a separate reading frame and enodes the reverse transcriptase (RT)/ribonuclease H (RNH) domains. ORF3 contains a tyrosine recombinase (YR) core domains at the carboxy terminus. ORF2 could be translated from a genomic DIRS-1 RNA as fusion to the YR domain by a +1 frameshift (not determined experimentally). Skipper-1 is a Ty3/*gypsy* retrotransposon that contains two ORFs flanked by identical LTRs. Skipper ORF1 codes for a GAG-like protein that includes a CX_2_CX_4_HX_4_C zinc finger-like motif [22]. ORF2 codes for a protease, RT, RNH, integrase (IN), and a chromo domain (CHD). The primer binding site (PBS) that is typical for Ty3/*gypsy* retrotransposons is replaced by a polypyrimidine sequence (PPy) downstream of the left LTR (Fig. 3). The *D. discoideum* Skipper-2 element is not listed in this figure because all copies are highly degenerated, but seems to have the same structural organization as Skipper-1. DGLT-A is a Ty3/*gypsy* retrotransposon that contains all protein functions in a single ORF [17]. The ORF contains a GAG-like protein with a CX_2_CX_4_HX_4_C zinc finger-like signature followed by RT, RNH, and integrase (IN) domains. Note that DGLT-A has no amino-terminal extension of the IN core domain and lacks a CHD. DGLT-A elements have a putative PBS 2 bp downstream of the left LTR (compare Fig. 3) and a polypurine tract (PPu) immediately upstream of the right LTR. Note that there are no Ty1/*copia*-like elements in the *D. discoideum* genome. The non-LTR retrotransposon family TRE separates into two subgroups, TRE5 and TRE3, named after their integration preferences upstream or downstream of tRNA genes [29]. All TRE elements contain two ORFs and have the same arrangement of protein domains in ORF2 in the order apurinic/apyrimidinic endonuclease (APE), RT domain, and a zinc-finger domain. The ORFs are flanked by short untranslated regions (UTR), and each element ends with a poly(A) tail of variable length. In contrast to the other TREs, TRE5-A has a modular structure determined by the duplication of the B-module [67]

The *D. discoideum* DIRS-1 element has inverted terminal repeats instead of LTRs and a complex arrangement of open reading frames (ORFs) that include an RT/RNH and a tyrosine recombinase (YR) instead of a canonical integrase (IN) [18, 19] (Fig. 1). DIRS-1 has a strong preference to integrate into existing DIRS-1 copies by a mechanism that probably involves YR-mediated homologous recombination [20]. Therefore, DIRS-1 forms complex clusters located near chromosome ends and contributes ~50 % of centromeric DNA of *D. discoideum* chromosomes [21].

DGLT-A and Skipper are related Ty3/*gypsy*-type LTR retrotransposons with strikingly different integration preferences. Skipper contains two ORFs coding for enzymatic activities required for retrotransposition arranged in the order RT-RNH-IN [22] (Fig. 1). Skipper is the prototype chromovirus in the *D. discoideum* genome as it contains a chromo domain (CHD) in the carboxy-terminal extension of the IN protein. The CHD may be responsible for targeting the element to centromeric regions where it contributes to ~10 % of centromer length [21]. It is known that centromeric DNA in *D. discoideum* has properties of heterochromatin including the presence of H3K9 methylation [23]. Retrotransposon CHDs may bind to methylated H3K9 and mediate their accumulation in heterochromatin [24], but it has not yet been determined experimentally whether Skipper is tethered to centromers via binding of its CHD to H3K9 methylation marks.

*D. discoideum* DGLT-A contains a single ORF and lacks a carboxy-terminal extension of the IN including a CHD as found in Skipper (Fig. 1). DGLT-A is related to Skipper but shows a completely different genomic distribution [17]; it does not accumulate in centromeric DNA but displays a strong preference to integrate within a window of 13–33 bp upstream of the mature coding sequences of tRNA genes [17]. The average distance of DGLT-A to the first nucleotide of a tRNA gene is 15 bp. This is remarkably similar to the integration preference of the yeast Ty3 element, considering that Ty3 inserts 1–4 bp upstream of the transcription start sites of tRNA genes [25], which is ~12 bp upstream of the first nucleotide of mature tRNAs [26]. It is not known whether the molecular mechanism of tRNA gene recognition of DGLT-A resembles that of Ty3, which identifies integration sites by binding of the IN to tDNA-bound transcription factor TFIIIB [27, 28].

The “tRNA gene targeted retroelements” (TREs) form two subfamilies of non-LTR retrotransposons (Fig. 1) that can be distinguished by phylogenetic analysis of their ORF2 proteins [17] and their integration preferences near tRNA genes [29]. TRE5 elements are strictly associated with regions ~50 bp upstream of tRNA genes, whereas TRE3 elements are always found ~100 bp downstream of tRNA genes. All TREs contain two ORFs. ORF1 proteins of TREs have no similarity among each other or with proteins of non-LTR retrotransposons such as the mammalian L1, in which the ORF1 protein is involved in binding the retroelement’s RNA as part of the pre-integration complex and contributes to the integration process [30, 31]. In *D. discoideum*, the ORF1 protein may be involved in the recognition of tRNA genes as integration sites by binding to subunits of RNA polymerase III transcription factor TFIIIB [32]. The TRE-encoded ORF2 proteins contain related apurinic/apyrimidinic endonuclease (APE) and RT domains (Fig. 1) that mediate retrotransposition.

It was of interest to trace the evolution of tRNA gene-associated mobile elements in social amoebae to understand how different tRNA gene-directed integration preferences emerged. In this study, we analyzed the annotated genomes of *D. discoideum*, *D. purpureum*, *D. lacteum*, *D. fasciculatum*, and *P. pallidum*, which represent the entire evolutionary history of social amoebae [16, 33, 34]. We found that the targeting of tRNA genes has independently developed at least six times through different mobile elements in the evolution of dictyostelids.

## Results

### Retrotransposons have excessively expanded in the *D. discoideum* genome

Hallmarks of the *D. discoideum* genome are the high gene density and the presence of retrotransposons that closely associate with tRNA genes, likely as a means to avoid insertional mutagenesis of host genes upon retrotransposition. This characteristic of the *D. discoideum* genome is similar to the yeast *Saccharomyces cerevisiae*, which has an even higher gene density than *D. discoideum* [35] and accommodates only retrotransposons that feature position-specific integration either near tRNA genes or in heterochromatin [36]. It has been of interest to compare integration preferences in yeast and dictyostelid genomes to evaluate whether tRNA gene-targeted integration presents an example of convergent evolution that enables mobile elements to settle in intergenic regions of compact genomes.

**Fig. 2:**
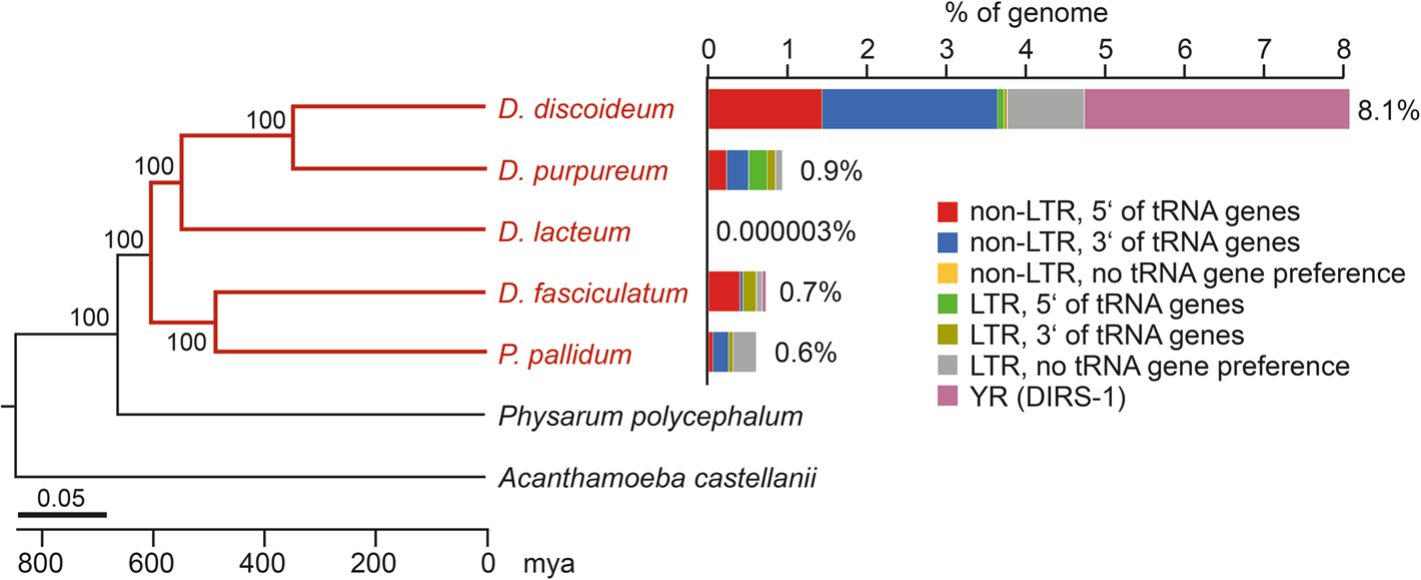
Phylogenetic relationships between dictyostelids. A genome-based phylogenetic tree was constructed on concatenated sequences of 32 orthologous proteins (redrawn from [13]). The retrotransposon content in each dictyostelid genome is plotted separated by the class of retrotransposon and integration preference near tRNA genes. YR: tyrosine recombinase retrotransposon (DIRS-1)

We evaluated retrotransposon families in the annotated genomes of *D. purpureum*, *D. lacteum, P. pallidum*, and *D. fasciculatum* in comparison with the model organism *D. discoideum*. The last common ancestor of all dictyostelids is estimated to date back approximately 600 million years and all examined species featured a long period of separate evolution [33] (Fig. 2), which must be considered when interpreting the relationships among transposable elements both within and outside the dictyostelids. We determined the retrotransposon contents of dictyostelid genomes by performing TBLASTX searches based on *D. discoideum* retrotransposon sequences of the tyrosine recombinase retrotransposon DIRS-1, the LTR retrotransposons Skipper and DGLT-A, and the non-LTR retrotransposons TRE5-A and TRE3-A (the structures of these elements are summarized in Fig. 1). The identified elements were reconstructed as consensus sequences. We also determined whether any of the identified retrotransposons may have a preference for integrating near tRNA genes by searching for tRNA genes within a distance of up to 3000 bp upstream and downstream of identified retroelements. A retrotransposon was considered to display active targeting to tRNA genes if several copies were found in a similar distance to tRNA genes. To ensure that we did not miss tRNA gene-targeting retrotransposons in this analysis, we performed a parallel search in which we first listed all tRNA genes of a given genome and then inspected 3000 bp upstream and downstream sequences for the presence of repetitive elements.

With the exception of *D. lacteum*, which has a particularly small and compact genome, all analyzed dictyostelids have comparable genome sizes of ~30 Mb and gene densities of close to 400 genes/Mb of genomic DNA (Additional file 1: Table S1). A notable difference between the genome of *D. discoideum* and any other examined species is the total retrotransposon content (Fig. 2, Additional file 1: Table S1). Whereas retrotransposons have expanded to 8 % of the *D. discoideum* genome, they have been kept below 1 % in other species.

DIRS-1 has strongly amplified in *D. discoideum* and constitutes 3.3 % of the genome in this organism [17]. The expansion of Skipper to 1.0 % of the *D. discoideum* genome may be linked to the amplification of DIRS-1, because both elements reside in centromeric DNA and may have adopted centromer function in this species [21]. Centromeric accumulation of DIRS-1 or Skipper is not observed in any other dictyostelid species except *D. fasciculatum*, which may form small centromeric DIRS clusters that contribute to only 0.1 % of genome size [33]. DIRS-1 is even missing in the assembled sequences of *P. pallidum* and *D. purpureum*. The data suggest that a putative centromere function of DIRS-1 (and Skipper) as observed in *D. discoideum* is deeply rooted in the social amoebae, even though the majority of species may have evolved deviant strategies to organize their centromeres without allowing the accumulation of selfish mobile elements in these regions.

A notable trend to increase the number of tRNA genes is observed in *D. discoideum* and *D. purpureum* relative to other dictyostelids (Additional file 1: Table S1). This observation is of interest considering that it may be easier for tRNA gene-targeting retrotransposons to expand if more potential safe integration sites are available. Whereas the tRNA gene-targeting DGLT-A-like retrotransposons are present in low copy numbers in all dictyostelds, a particularly strong amplification in *D. discoideum* relative to other species is observed in the TRE family (Fig. 2, Additional file 1: Table S1). Such expansion is not observed in the genome of *D. purpureum*, which has a comparable amount of tRNA genes. Thus, targeting preference near tRNA genes does not necessarily favor the amplification of position-specific integrating elements to high copy numbers under the repressive conditions that prevail in most host cells.

### Dictyostelid LTR retrotransposons comprise related families with different tRNA gene-targeting strategies

As previously noted by Malik et al. [7], IN domains of Ty3/*gypsy*-type retrotransposons frequently contain carboxy-terminal extensions including a distinctive GPY/F motif at the end of the IN core followed by relatively unconserved domains of various sizes that may harbor a chromo domain (CHD). *D. discoideum* DGLT-A has a small IN extension of 32 amino acids, whereas Skipper has a long IN extension of 183 amino acids that contains a CHD. In the analysis of dictyostelid genomes described below, we found that all new identified LTR retrotransposons have the Ty3/*gypsy*-type structure including a conserved GPY/F motif (Additional file 1: Figure S1). For convenience, we call retrotransposons “Skipper” if they contain a CHD in the carboxy-terminal extension of the IN domain and “DGLT-A” if a CHD is lacking.

Twenty insertions of DGLT-A are detectable in the *D. discoideum* genome, eleven of which are solo LTRs that were formerly described as “H3R” elements located upstream of tRNA genes [37]. None of the remaining nine DGLT-A sequences are full-length and refer to the derived consensus of this element (Table 1). This suggests that the DGLT-A population may no longer be able to amplify in the *D. discoideum* genome, even though all ORF domains are transcribed in growing *D. discoideum* cells (T.W., unpublished observation).

**Table 1:** Overview of dictyostelid retrotransposon properties and integration preferences

Name	Consensus length (bp)	LTR length (bp)	Copy number in genome ^a^			tRNA gene-specific	Distance to tRNA gene (bp)	
			total	full length ^b^	solo LTR		5’ of tDNA (%) ^c^	3’ of tDNA (%) ^c^
LTR retrotransposons								
Dd-Skipper-1	6998	390	60	2	10	no	–	–
Dp_Skipper-1	7485	388	12	1	6	no	–	–
Dl_Skipper-1	4763	251	7	2	2	no	–	–
Pp_Skipper-1	5589	226	14	1	10	yes	–	–
Df_Skipper-1	5120 ^d^	n.d. ^e^	5	0	n.d. ^e^	no	–	–
Pf_Skipper-1.1	5296	259	6	1	0	no	–	–
Pf_Skipper-1.2	6983	382	3	0	1	no	–	–
Pf_Skipper-1.3	7081	363	7	1	6	no	–	–
Dd-Skipper-2 ^f^	6178	208	8	0	0	no	–	8–23 (4)
Dp_Skipper-2	5676	315	23	3	5	yes	–	7–133 (5)
Pp_Skipper-2	3675 ^d^	n.d. ^e^	9	0	n.d. ^e^	yes	–	54–136 (9)
Df_Skipper-2	5708	312	12	7	5	yes	–	26–97 (11)
Dd_DGLT-A	5054	265	20	0	5	yes	13–33 (18)	–
Dp_DGLT-A.1	5436	492	15	1	9	yes	13–16 (6)	–
Dp_DGLT-A.2	6114	389	9	2	5	yes	15 (1)	–
Dp_DGLT-A.3	5589	563	8	1	6	yes	10–11 (2)	–
Dp_DGLT-A.4 ^g^	3447 ^d^	206	30	0	20	yes	16–34 (4)	–
Dp_DGLT-A.5 ^g^	3440 ^d^	354	32	0	32	yes	10–19 (15)	–
Dl_DGLT-A.1	4895	163	10	1	4	yes	63–64 (4)	–
Dl_DGLT-A.2	5112	206	7	1	2	yes	55–65 (2)	–
Pp_DGLT-A.1	7295	601	23	1	13	no	–	–
Pp_DGLT-A.2	6160	393	12	2	6	no	–	–
Pp_DGLT-A.3	5942	212	11	2	3	no	–	–
Pp_DGLT-A.4 ^g^	3650 ^d^	n.d. ^e^	10	0	n.d. ^e^	yes	14–24 (3)	–
Pf_DGLT-A	8367	168	2	1	1	no	–	–
Non-LTR retrotransposons								
Dd_TRE3-A	5229	–	67	13	–	yes	–	14–228 (60)
Dd_TRE3-B	5279	–	43	9	–	yes	–	34–188 (39)
Dd_TRE3-C	4734	–	29	2	–	yes	–	14–305 (29)
Dd_TRE3-D	1559 ^d^	–	11	0	–	yes	–	49–285 (11)
Dp_TRE3-A	5150	–	56	2	–	yes	–	69–161 (18)
Dp_TRE3-B	5210	–	9	2	–	yes	–	98–154 (2)
Dp_TRE3-C	1620 ^d^	–	37	0	–	yes	–	67–450 (15)
Dl_TRE3-A	4386	–	17	2	–	yes	–	23/87 (7)
Pp_TRE3-A	4515	–	35	4	–	yes	–	26–138 (10)
Pp_TRE3-B	4741	–	38	2	–	yes	–	57–151 (11)
Df_TRE3-A	1867 ^d^	–	14	0	–	yes	–	29–404 (11)
Dd_TRE5-A	5647	–	102	5	–	yes	37–90 (98)	–
Dd_TRE5-B	5971	–	25	1	–	yes	34–82 (25)	–
Dd_TRE5-C	879 ^d^	–	18	0	–	yes	38–95 (18)	–
Dl_TRE5-A ^h^	7405	–	30	1	–	no	–	–
Pp_TRE5-A	1169 ^d^	–	21	0	–	yes	38–74 (12)	–
Df_TRE5-A.1	2587 ^d^	–	56	1	–	yes	45–88 (20)	–
Df_TRE5-A.2	1275 ^d^	–	20	0	–	yes	31–98 (18)	–
Df_TRE5-A.3	2941 ^d^	–	7	0	–	yes	39–67 (6)	–
Df_TRE5-B	1534 ^d^	–	27	0	–	yes	44–90 (9)	–
Dp_NLTR-A	7438	–	28	1	–	yes	2–6 (16)	–
Pp_NLTR-B	5550 ^d^	–	3	0	–	yes	39–64 (3)	–
Pp_NLTR-C	3536 ^d^	–	12	1	–	no	–	–

^a^Total copy numbers refer to both full-length and partial sequences

^**b**^Full-length copies with intact open reading frames

^c^Distances are listed only for retrotransposons found in the direct neighborhood of tRNA genes; in cases where other tRNA gene-specific retrotransposons have integrated at the same tRNA gene and therefore upstream of a previously inserted element, distances of the original insertion to the target could not be determined. The number of elements used for determination of target distances are shown in parentheses

^d^No full-length consensus available

^e^No LTR sequences detectable

^f^Previous name DGLT-B (GenBank AF474004) [17]

^g^No ORFs for phylogenetic analysis; classification as DGLT-A according to integration preference

^h^Classified as TRE5 by similarity of RT sequence (compare Fig. 5)

The *D. purpureum* genome contains three related DGLT-A elements, of which each retained at least one retrotransposition-competent copy. *D. purpureum* DGLT-As have the same structure and display the same target preference 13–16 bp upstream of tRNA genes as the prototype DGLT-A of *D. discoideum* (Table 1). Two related full-length DGLT-A elements were detected in the *D. lacteum* genome. These elements also display integration preference upstream of tRNA genes (Table 1). The *P. pallidum* genome contains four related DGLT-A elements. Of these, Pp_DGLT-A.1, Pp_DGLT-A.2, and Pp_DGLT-A.3 comprise a population of elements with intact open reading frames and probable retrotransposition competence. Unlike other DGLT-As, Pp_DGLT-As contain long carboxy-terminal IN extensions of 264–333 amino acids but no detectable CHDs. The IN extensions in *P. pallidum* DGLT-A elements are poorly conserved among each other and do not show similarity with other retrotransposons such as dictyostelid Skipper or yeast Ty1 and Ty3 elements. Notably, Pp_DGLT-A.1, Pp_DGLT-A.2, and Pp_DGLT-A.3 do not show a preference to integrate near tRNA genes. However, we detected a partial sequence of a fourth DGLT-A in the *P. pallidum* genome (Pp_DGLT-A.4) that is related to the other *P. pallidum* DGLT-As by phylogenetic analysis of the intact RT and RNH domains (data not shown) and its preference to integrate 14–25 bp upstream of tRNA genes (Table 1). This suggests that the tRNA gene preference of DGLA-A has also been established in the *P. pallidum* genome but was lost in some DGLT-A lineages. The conclusion from this observation is that tRNA gene targeting by DGLT-As was established in the earliest diverged species of Dictyostelia.

The Skipper-1 element of *D. discoideum* is 34 % identical with DGLT-A in the RT-RNH-IN core domains but does not display integration specificity at tRNA genes. Instead, the approximately 60 Skipper copies are highly enriched in centromeric transposon clusters [21]. Two Skipper copies can be identified in the *D. discoideum* genome that have intact open reading frames and may be retrotransposition-competent.

The *D. purpureum* genome contains two related Skipper elements. Dp_Skipper-1 is highly similar to Dd_Skipper-1 and does not show association with tRNA genes. In contrast, Dp_Skipper-2, of which three intact copies exist in the *D. purpureum* genome, is found within a range of 7–133 bp downstream of tRNA genes (Table 1). This integration preference of an LTR retrotransposon had not been observed before. However, in the course of this study, we re-evaluated the previously described DGLT-P element of *D. discoideum* [17] and detected a CHD in the highly degenerated ORF of this element and surprisingly noticed that 4 of 8 copies of this element are located in a range of 8–23 bp downstream of tRNA genes. We therefore renamed DGLT-P “Dd_Skipper-2”. Interestingly, a Skipper-like element with target preference downstream of tRNA genes was also detected in the *D. fasciculatum* genome. The Df_Skipper-2 element was found inserted 26–97 bp downstream of tRNA genes, whereas a related Df_Skipper-1 element does not display target specificity (Table 1). The *P. pallidum* genome also contains two related Skipper-like elements, of which the Skipper-2 is found within a window of 54–136 bp downstream of tRNA genes. The *D. lacteum* genome contains one intact copy of a Skipper element (Dl_Skipper-1) that is not associated with a tRNA gene. In summary, it seems that Skipper elements diverged into two subfamilies, of which one (Skipper-2) developed a previously unnoticed preference to integrate downstream of tRNA genes. This is interesting because integration preference for the same region was also invented by the unrelated non-LTR retrotransposons of the TRE3 family described later.

**Fig. 3:**
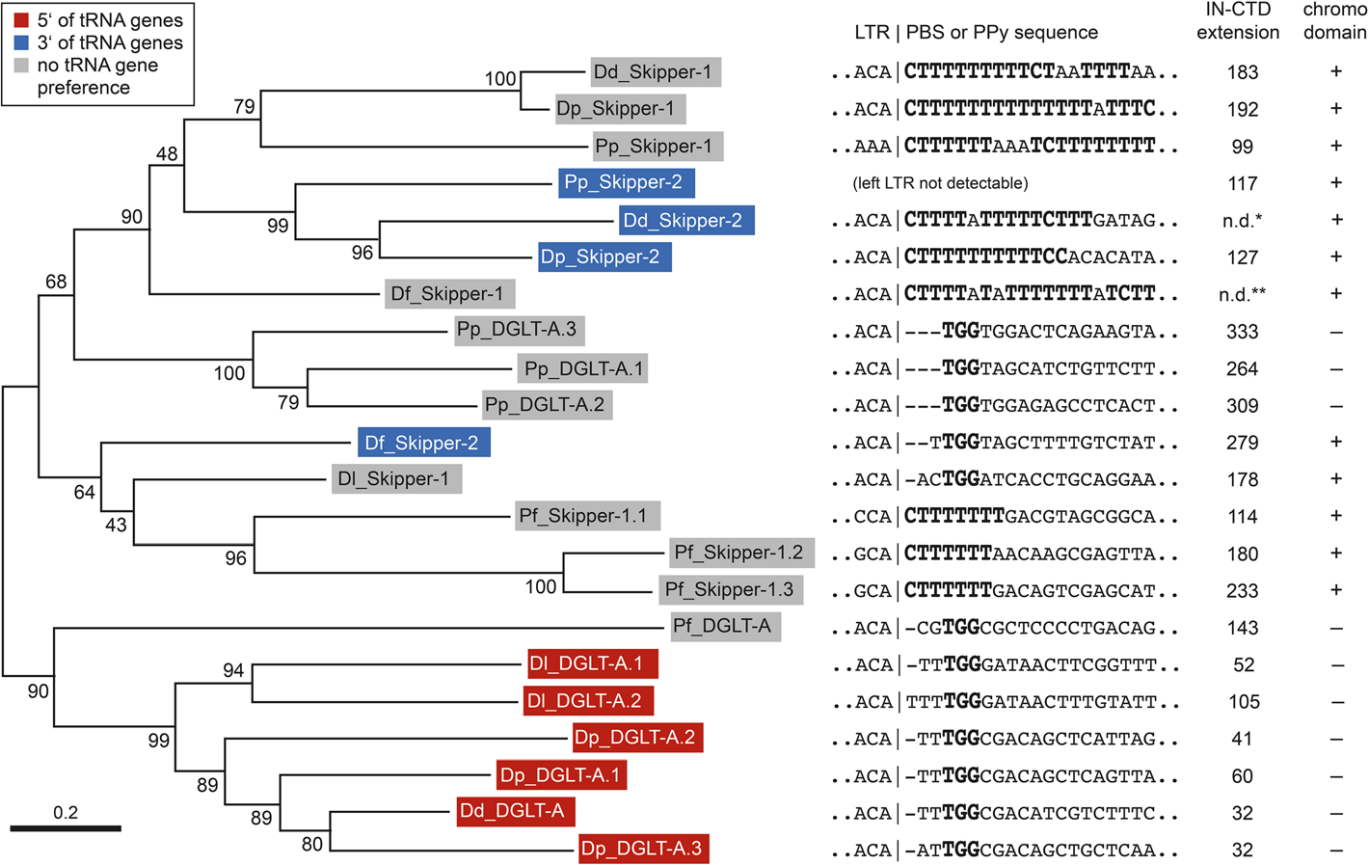
Phylogeny of dictyostelid LTR retrotransposons. Alignment of the concatenated core domains of RT, RNH, and IN was generated with ClustalX and analyzed using the Maximum Likelihood method. Numbers next to each node indicate bootstrap values as percentages out of 1000 replicates. All positions containing gaps and missing data were eliminated. The tree is drawn to scale with branch lengths measured in the number of substitutions per site. Analysis of the data using the Neighbor Joining method produced a slightly different tree topology (see Additional file 1: Figure S3). Retrotransposons integrating upstream and downstream of tRNA genes are indicated in red and blue boxes, respectively. The sequence immediately downstream of the left LTR in each retrotransposon is shown, with the TGG signature of a canonical primer binding site (PBS) or the polypyrimidine tract (PPy) highlighted in bold. The lengths of the carboxy-terminal extensions of IN domains in individual retrotransposons are indicated and were calculated from the conserved GPY/F motifs to the carboxyl end. It is also indicated whether the IN extensions contain a chromo domain. Dd: *D. discoideum*; Dp: *D. purpureum*; Dl: *D. lacteum*; Df: *D. fasciculatum*; Pp: *P. pallidum*; Pf: *Protostelium fungivorum*; IN-CTD: carboxy-terminal extension of integrase. n.d.: not determined (*: ORF degenerated; **: length of extension undetermined because LTR is missing)

Phylogenetic analyses based on alignments of the concatenated RT-RNH-IN core domains of all LTR retrotransposons (Additional file 1: Figure S2) support the division of these elements into DGLT-A and Skipper families but also reveal interesting differences in the evolution of these elements (Fig. 3, Additional file 1: Figure S3). For example, DGLT-A elements from *D. discoideum*, *D. purpureum*, and *D. lacteum* form a robust group of elements that share an integration preference upstream of tRNA genes. However, DGLT-A.1, DGLT-A.2, and DGLT-A.3 of *P. pallidum* clustered with Skipper elements, which was unexpected because *P. pallidum* DGLT-A.4 (not included in the phylogenetic analysis shown in Fig. 3) showed the DGLT-A-typical integration preference upstream of tRNA genes. On the other hand, the *P. pallidum* DGLT-As that clustered among Skipper elements have long IN extensions reminiscent of Skipper elements, but they lack a detectable CHD.

**Fig. 4:**
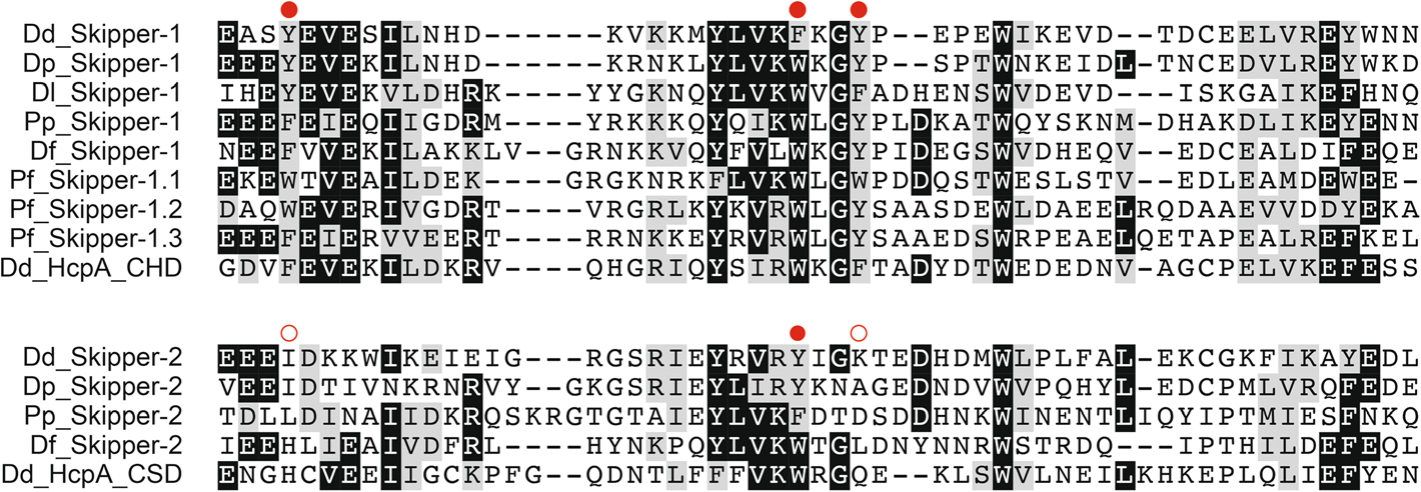
Alignments of chromo domains in dictyostelid and protostelid Skipper retrotransposons. Alignments were generated with ClustalX. Shading is to a 50 % consensus and was generated with BoxShade. Black boxes indicate invariant amino acids, and gray boxes represent similar amino acids. The corresponding sequences of the chromo domain (CHD) and the “shadow” chromo domain (CSD) of *D. discoideum* heterochromatin protein 1 (HcpA) [38] are shown for comparison. Red dots depict aromatic cage residues present in canonical chromo domains [39]. The alignment is separated into retrotransposons containing canonical (group I) chromo domains (Skipper-1 elements) and group II chromo domains (Skipper-2 elements) in which the first and third aromatic amino acid of the cage are diverged (indicated by open circles). Dd: *Dictyostelium discoideum*; Dp: *D. purpureum*; Dl: *D. lacteum*; Pp: *Polysphondylium pallidum*; Df: *D. fasciculatum*; Pf: *Protostelium fungivorum*

The phylogenetic analysis presented in Fig. 3 implies a further separation of Skipper elements into two subfamilies: Skipper-1 without target preference and Skipper-2 that integrate downstream of tRNA genes. Notably, all Skipper elements contain carboxy-terminal extensions of the IN core ranging from 99 to 192 amino acid that include distinctive CHDs. The CHDs of Skipper elements are compared in Fig. 4 with the CHD and chromo shadow domain (CSD) of *D. discoideum* heterochromatin protein 1 (HP1), which is known to bind to heterochromatin via its CHD interacting with methylated lysine-9 of histone H3 (H3K9) while its CSD comprises a dimerization domain [38]. Each Skipper-1 retrotransposon contains a canonical HP1-like CHD that has three conserved aromatic amino acids known to build a “cage” responsible for the binding to methylated H3K9 [39] (Fig. 4). Whether CHDs of Skipper-1 elements indeed bind to methylated histone H3 lysine 9 marks and tether the elements to centromeric regions has not yet been experimentally tested. Gao et al. [24] analyzed CHDs of various LTR retrotransposons and concluded that they can be grouped into “canonical” CHDs (group I CHDs) and derivatives that lack the first and usually also the third of the aromatic cage residues (group II CHDs). Interestingly, all Skipper-2 elements have diverged exactly the same aromatic cage residues in their CHDs, which in fact resembles the HP1 CSD (Fig. 4). This suggests that CHDs of Skipper-2 elements may be in the process of functional degeneration or, more intriguing, have been modified to shift the integration behavior of these elements to new locations outside of heterochromatin. In this regard, it is of note that Skipper-2 elements apparently evolved a new integration preference downstream of tRNA genes in intergenic regions as described above.

### Many Skipper elements have lost the canonical primer binding site to initiate reverse transcription

A primer binding site (PBS) located immediately downstream of the U5 sequence in the left LTR is required to initiate minus-strand strong-stop cDNA synthesis in most Ty3/*gypsy* retrotransposons [40, 41]. The PBS usually presents a TGG trinucleotide signature as a complement of the CCA 3’ end of a host tRNA that is used as primer to initiate reverse transcription. In *D. discoideum* DGLT-A, the sequence TGGCGACATCGTCTTTC is located 2 bp downstream of the left LTR (Fig. 3), but no tRNA or any other genomic sequence complementary to the PBS could be identified in the *D. discoideum* genome as a potential primer for reverse transcription of DGLT-A.

In contrast to DGLT-A, most elements classified as Skipper according to the presence of a CHD have apparently replaced the canonical PBS with degenerate polypyrimidine (PPy) sequences (Fig. 3) that suggest a non-canonical mechanism of reverse transcription priming. Interesting exceptions are found in Skipper-like elements from *D. lacteum* and *D. fasciculatum*: Dl_Skipper-1 has a CHD indicative of Skipper, but contains a PBS typical for DGLT-A. Likewise, Df_Skipper-2 contains a DGLT-A-type PBS and a group II CHD. At least seven intact copies Df_Skipper-2 suggest that the element is retrotransposition-competent; all copies are found within a window of 26–97 bp downstream of tRNA genes (Table 1).

### The Skipper and DGLT-A families originated before the evolution of dictyostelds

The long independent evolutionary history of Amoebozoa makes it difficult to trace the origin of DGLT-A- and Skipper-like retrotransposons and the invention of their tRNA gene targeting mechanisms outside the Dictyostelia. The recently obtained genome sequence of a *Protostelium* species (F.H., T.W., G.G., manuscript in preparation) is helpful, because even though Protostelia are polyphyletic [42], they are considered closer related to the monophyletic Dictyostelia than other amoebozoan species sequenced so far such as *Acanthamoeba castellanii* or *Physarum polycephalum*. The genome of the sequenced protostelid, *P. fungivorum*, contains one DGLT-A-like and three Skipper-like elements (Table 1). The Skipper-like elements contain the typical PPy signature downstream of the left LTR (Fig. 3) and a canonical CHD downstream of IN (Fig. 4), supporting the hypothesis that the Skipper-type LTR retrotransposons arose outside the Dictyostelia. Although the gene density of the *P. fungivorum* genome is comparable with the dictyostelids, none of the *P. fungivorum* DGLT-A- or Skipper-like elements has developed integration preferences for tRNA genes. Because the absence of targeting preferences of LTR retrotransposons in this particular *Protostelium* isolate is not an argument for the *de novo* invention of such a specificity in dictyostelids, the origin of tRNA gene targeting in dictyostelid genomes remains a mystery until more amoebozoan genomes have been sequenced.

### Dictyostelid non-LTR retrotransposons evolved four different tRNA gene-targeting strategies

In the *D. discoideum* genome, TRE elements can be distinguished between the TRE5 and TRE3 subfamilies according to their exclusive integration behavior [17]. TRE elements comprise 3.7 % of the *D. discoideum* genome, with TRE5-A and TRE3-A contributing the majority of individual copies (Table 1). In *D. discoideum*, 61 % of tRNA genes are associated with at least one TRE element (Additional file 1: Table S2), and 13 % of tRNA genes have been targeted by both TRE3 and TRE5.

**Fig. 5:**
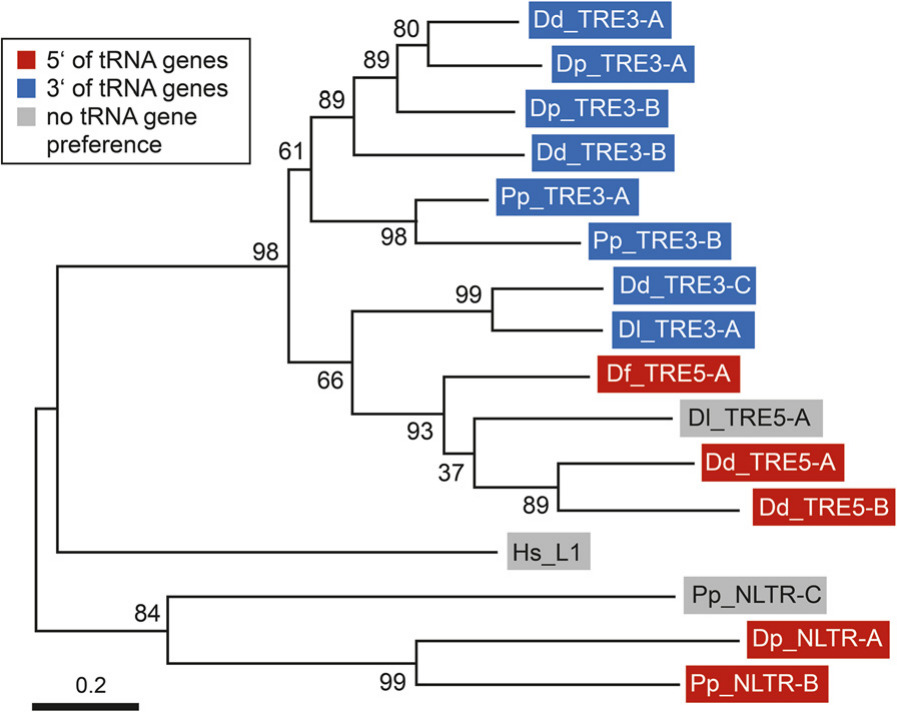
Phylogenetic analysis of dictyostelid non-LTR retrotransposons. Alignment of RT domains was generated with ClustalX and analyzed using the Maximum Likelihood method. All positions containing gaps and missing data were eliminated. The tree is drawn to scale with branch lengths in the same units as those of the evolutionary distances used to infer the phylogenetic tree. There were a total of 227 amino acid positions in the final dataset. Bootstrap support (percentage from 1000 trials) is indicated next to each node

We considered newly discovered non-LTR retrotransposons in dictyostelid genomes as TRE5-like and TRE3-like if they were found upstream and downstream of tRNA genes, respectively, at similar distances as in the *D. discoideum* genome. We examined the evolution of TRE5- and TRE3-like elements using the complete ORF2 sequences of *D. discoideum* TREs as query sequences in TBLASTX searches. We identified TRE5- and TRE3-like sequences in *D. lacteum*, *D. fasciculatum* and *P. pallidum*, whereas *D. purpureum* contains only TRE3-like sequences (Table 1). Alignments of the conserved RT domains (Additional file 1: Figure S4) and phylogenetic analyses (Fig. 5) support the evolution of TRE5 and TRE3 in separate subfamilies with the exception of Dd_TRE3-C, which appeared to be more related to TRE5 elements than to TRE3 elements in these analyses. This grouping of Dd_TRE3-C is likely caused by the relatively short RT amino acid sequences used in this analysis because this element clusters robustly with the other TRE3 elements when examining the complete ORF2 sequences [17]. Phylogenetic analyses on the entire ORF2 proteins across species was not feasible in this study because complete elements could not be reconstructed in all genomes. TRE-like retrotransposons were found to be associated with tRNA genes at locations typical for *D. discoideum* TRE5 and TRE3 elements (Table 1), suggesting that this type of integration behavior is deeply rooted within the dictyostelids. TRE-like elements have not been identified in the genomes of distantly related amoebozoans such as *Physarum polycephalum* and *Acanthamoeba castellanii* and are also absent in the recently sequenced isolate of *Protostelium fungivorum*. Therefore, the origin of the last common ancestor of the TREs (including the evolution of their unique integration preferences) remains to be determined.

**Fig. 6:**
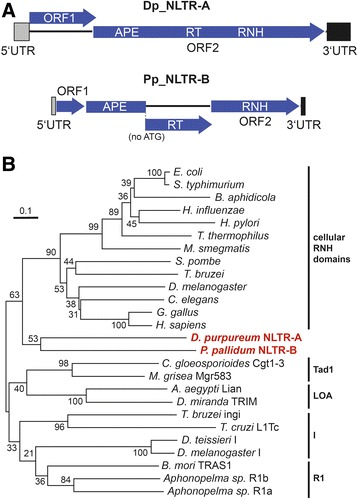
Phylogenetic analysis of RNH domains in dictyostelid NLTR elements. **a** Schematic presentations of the structures of *D. purpureum* NLTR-A and *P. pallidum* NLTR-B. See Fig. 1 for abbreviations. **b** Sequences of RNH domains were analyzed using the Neighbor Joining method. All positions containing gaps and missing data were eliminated. The tree is drawn to scale, with branch lengths in the same units as those of the evolutionary distances used to infer the phylogenetic tree. Numbers next to each node indicate bootstrap values as percentages out of 1000 replicates [68]. Analysis of the data with the Maximum Likelihood method produced the same tree topology with slightly lower bootstrap values. The tree is drawn to scale, with branch lengths measured in the number of substitutions per site. The tree was rooted on cellular RNH domains. Sequences used for alignment with *D. purpureum* NLTR-A and *P. pallidum* NLTR-B were chosen according to a previous phylogenetic analysis by performed by Malik et al. [11]: *Drosophila miranda* TRIM (X59239), *Aedes aegypti* Lian (U87543), *Colletotrichum gloeosporioides* Cgt1-3 (L76169), *Magnaporthe grisea* Mgr583 (AF018033), *Trypanosoma bruzei* ingi (X05710). *Trypanosoma cruzi* L1Tc (X83098), *Drosophila teissieri* I (M28878), *Drosophila melanogaster* I (M14954), *Bombyx mori* TRAS (GenBank D38414), and *Aphonopelma sp.* R1a (AF015489) and R1b (AAB94039). Cellular RNH domains used as outgroups were follows: *Escherichia coli* (P00647), *Salmonella typhimurium* (P23329), *Buchnera aphidicola* (Q08885), *Haemophilus influenzae* (P43807), *Helicobacter pylori* (P56120), *Thermus thermophilus* (P29253), *Mycobacterium smegmatis* (Q07705), *Schizosaccharomyces pombe* (AAC04366). *Trypanosoma bruzei* (AAC47537), *Drosophila melanogaster* (AAC47810), *Caenorhabditis elegans* (AAA83453). *Gallus gallus* (BAA05382), and *Homo sapiens* (CAA11835)

We detected new non-LTR retrotransposons in the genomes of *D. purpureum* and *P. pallidum* that we tentatively named “non-LTR” (NLTR) elements because they are only distantly related to TRE elements based on phylogenetic analysis of RT domains (Fig. 5, Additional file 1: Figure S4). *D. purpureum* NLTR-A and *P. pallidum* NLTR-B are 38 % identical to each other in their RT domains and are characterized by an RNH domain located downstream of the RT (Fig. 6). Intriguingly, Dp_NLTR-A and Pp_NLTR-B developed different target preferences upstream of tRNA genes (Table 1). Dp_NLTR-A was found 2–6 bp upstream of the first nucleotide of the mature coding sequence of the targeted tRNA gene, which represents an as-yet unobserved integration specificity, whereas Pp_NLTR-B was found at similar positions as TRE5 elements ~50 bp upstream of tRNA genes. *P. pallidum* NLTR-C was identified as a partial sequence that contains an RT domain. This element is only distantly related to Dp_NLTR-A and Pp_NLTR-B (~26 % sequence identity in the RT domain) and does not show association with tRNA genes. Phylogenetic analysis based on RT domains considering all major subgroups of non-LTR retrotransposons [11] failed to place the Dp_NLTR-A and Pp_NLTR-B elements in any of the subfamilies of non-LTR retrotransposons that are known to harbor an RNH domain (Additional file 1: Figure S5). A phylogenetic evaluation of RNH domains of non-LTR retrotransposons based on alignments previously proposed by Malik et al. [11] confirmed that Dp_NLTR-A and Pp_NLTR-B may form a separate group within the supergroup of non-LTR retrotransposons (Fig. 6; Additional file 1: Figure S6). The Pp_NLTR-C RT sequence aligned best with subgroup R4 elements; however, this grouping could not be evaluated further because no restriction enzyme-like endonuclease domain, which is typically located downstream of RTs in R4-like elements [11], was included in the partial Pp_NLTR-C sequence.

## Discussion

### Convergent evolution of integration site selection in compact genomes

Integration behaviors of retrotransposons residing in compact genomes of different organisms show parallels that suggest strong convergent pressures to avoid insertional mutagenesis of genes and to preserve genome stability of the host. The haploid state of dictyostelid genomes may further increase the selection pressure on mobile elements because the disruption of an essential host gene in the absence of a second compensatory allele would ultimately eliminate the parasite along with its host. In dictyostelids, two principally different strategies have emerged to counter this selection pressure: (i) integration in gene-poor regions of centromeric DNA, which restricts mobile elements to certain spots of repetitive DNA in the host genome and (ii) the targeting of tRNA genes, which not only appears to represent the prime “safe sites” to integrate in gene-rich regions but also enables mobile elements to settle anywhere in the genome due to the multicopy nature of their targets and dispersal on all chromosomes.

In *S. cerevisiae*, the Ty1/*copia*-type retrotransposon Ty5 is tethered to regions of silent chromatin via direct protein interactions of Ty5 IN with heterochromatin-associated protein Sir4 [43]. There are no Ty1/*copia*-type retrotransposons found in dictyostelid genomes, but Skipper and DIRS-1 elements accumulate in centromer regions that are organized as heterochromatin. The heterochromatin-targeting mechanisms developed by Skipper and DIRS are different from each other and from Ty5. As we discuss in more detail below, Skipper elements are likely tethered to centromeres via interactions between their chromo domains and histone methylation marks that are characteristic for heterochromatin. The DIRS-1 element is special because it encodes a tyrosine recombinase (YR) instead of a canonical IN and is thought to generate circular retrotransposition intermediates that are probably targeted to centromers via YR-mediated homologous recombination into pre-existing DIRS-1 copies [18, 20].

**Fig. 7:**
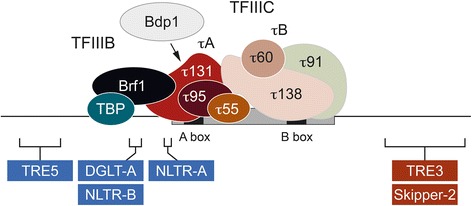
Summary of integration sites near tRNA genes in dictyostelid genomes. The topology of RNA polymerase III transcription factors TFIIIC and TFIIIB on a tRNA gene is shown as deduced by Male et. al. [69]. The composition of TFIIIB in three subunits is inferred by the presence of orthologs of TBP, Brf1 and Bdp1 in all dictyostelid species. TFIIIC is a six-subunit factor that consists of two subcomplexes, τA and τB [69]. Note that TFIIIB subunit Bdp1 may enter the transcription complex only transiently by displacing TFIIIC subcomplex τB [69]. In dictyostelid genomes only the most conserved TFIIIC subunits τ131 (TFC4) and τ95 (TFC1) can be identified by homology to either yeast or human orthologs. The schematic is not drawn to scale. The tRNA gene, including its internal regulatory sequences (A box and B box), is indicated as a gray bar. Integration windows in tRNA gene-flanking regions of six different dictyostelid retrotransposon families are indicated. Note that DGLT-A and NLTR-B belong to different retrotransposon classes and therefore independently developed a similar integration behavior upstream of tRNA genes. The same is true for TRE3 and Skipper-2 elements, which target similar regions downstream of tRNA genes

The targeting of tRNA genes as presumed safe integration sites has been independently developed at least six times by retrotransposons during dictyostelid evolution (summarized in Fig. 7) and at least twice in the yeast *S. cerevisiae*. Ty1 and Ty3 elements, which belong to different classes of LTR retrotransposons, obviously evolved different mechanisms for tRNA gene recognition. Ty1 integrates within a window of ~750 bp upstream of tRNA genes that is defined by nucleosome positioning [44, 45] and direct interactions between Ty1 IN and RNA polymerase III subunits [46, 47]. A Ty1-like integration behavior of retrotransposons has not been observed in dictyostelid genomes. In contrast, there is a striking similarity of integration site selection between Ty3 and dictyostelid DGLT-A elements. Ty3 targets the entire RNA polymerase III transcriptome of *S. cerevisiae* [48], particularly in regions 1–4 bp upstream of the transcription start sites of tRNA genes (that is, ~15 bp upstream of the first nucleotide of the mature tRNA) [25]. This target preference is mediated by an interaction between Ty3 IN and subunits of RNA polymerase III transcription factor TFIIIB [27]. In most dictyostelids evaluated in this study, DGLT-A elements have conserved an integration preference approximately 15 bp upstream of tRNA genes. It would be interesting to determine whether DGLT-A elements use the same molecular interactions to recognize RNA polymerase III-transcribed genes as Ty3 or whether selection pressure to avoid gene mutagenesis has generated other solutions to the problem of targeting tRNA gene-upstream regions in different lineages of retrotransposon evolution.

The targeting of tRNA genes by TRE elements is unique to and deeply rooted in the dictyostelids. Although TRE5 and TRE3 elements evolved from a common ancestor [17] that most likely dates back before dictyostelid evolution, these elements developed strikingly different integration preferences and thus use different molecular mechanisms for target recognition. The integration window preferred by TRE3-A elements strikingly overlaps with the integration profile displayed by the unrelated Skipper-2 elements, suggesting that a region ~100 bp downstream of tRNA genes is accessible for retrotransposons to develop harmless integration strategies in compact genomes. The targeting mechanisms of TRE elements have been investigated experimentally in some detail only in the TRE5-A element, which requires intact B boxes in targeted tRNA genes and probably DNA-bound RNA polymerase III transcription complexes for integration [49]. In vitro data suggest interaction between TRE5-A ORF1 protein and TFIIIB subunits during the integration process [32], which in turn is a remarkable parallel to target recognition by the otherwise unrelated yeast Ty3 element.

Interestingly, high copy numbers of retrotransposons were only found in *D. discoideum* and not in other dictyostelid genomes. Our data suggest that *D. discoideum* is different from the other investigated dictyostelids in that it was specifically affected by an unknown selection pressure that either demanded or coincidentally enabled a burst of retrotransposon expansion. It seems unlikely that the propagation of the sequenced laboratory strain AX4 for about four decades has caused this retrotransposon expansion, because Southern blot data on genomic DNA of the parent strain NC4 probing for TRE5-A and TRE3-A indicated similarly high copy numbers of both elements (T.W., unpublished data). It is conceivable that *D. discoideum* has evolved to enable DIRS-1 amplification in centromeres to serve the organism as a substrate for kinetochore complex formation. The tRNA gene-targeting retrotransposons may have profited from this selection and, as a consequence, expanded throughout the genome. However, cells affected in such a manner may have been negatively selected even if there was no direct damage to genes because the haploid genome is particularly vulnerable to non-allelic recombinations forced by the accumulation of repetitive DNA. This consideration may explain why the targeting of tRNA genes by TRE elements achieved a steady state at approximately 60 % saturation of tRNA gene loci.

### Skipper elements may use unconventional priming of reverse transcription

During the analysis of dictyostelid genomes, the question arose as to whether Skipper elements use a novel mechanism of reverse transcription initiation. Many retroviruses and LTR retrotransposons use cellular tRNAs as primers to initiate minus-strand strong-stop cDNA synthesis [40, 41]. These elements are characterized by a typical TGG trinucleotide signature located a few base pairs downstream of the left LTR that presents the complement of the CCA 3’-end of tRNA primers. All identified DGLT-A elements have this typical TGG motif 2 bp downstream of the left LTR (Fig. 3), but no cellular tRNAs could be identified that may be used as primers for cDNA synthesis. In contrast, most Skipper elements lack the TGG motif and instead contain a degenerate polypyrimidine (PPy) stretch. Although this characteristic feature of Skipper elements could be traced to a *Protostelium* species suggesting a root outside the dictyostelids, it has not been found in other organisms to the best of our knowledge. Some LTR retrotransposons lacking the TGG signature are assumed to use self-priming to initiate reverse transcription [50]. In such elements, RNA sequences located in the left LTR at the 5’ ends of the retrotransposon transcripts loop back to the region immediately downstream of the LTR and prime reverse transcription [51]. Regarding the Skipper elements, no such complementary regions in the left LTRs are present, suggesting that a novel type of self-priming may be involved. It is unlikely, however, that a “simple” poly(A) stretch somewhere in the Skipper sequence is used in a self-priming process because the PPy sequences in all Skipper elements bear a characteristic C nucleotide facing the orientation of minus-strand cDNA synthesis (Fig. 3).

### Dictyostelid Skipper elements are typical chromoviruses

In *D. discoideum*, DIRS-1 and Skipper elements form large clusters at the nuclear periphery during interphase that splits into six distinct spots during mitosis representative of the centromeric DNA of the six chromosomes [23]. Interestingly, the clustering of retrotransposons in heterochromatic regions has also been reported in fungal genomes such as that in *Magnaporthe grisea*, an organism with a similarly high gene density as dictyostelids [52]. This type of retrotransposon clustering appears to differ from the targeting of yeast Ty5 to heterochromatin and likely involves interactions of chromo domains located downstream of IN domains in Ty3/*gypsy*-type retrotransposons with heterochromatin marks. Similar to DIRS-1, Skipper-1 from *D. discoideum* has been shown to co-localize with sites of H3K9me2 methylation [23] and binding sites of CenH3, a marker for centromeric heterochromatin [53]. DIRS-1 and Skipper also co-localize with heterochromatin protein 1 (HP1; HcpA), which is recruited to centromeric heterochromatin through the binding of its chromo domain (CHD) to H3K9me2 marks [38]. Skipper shows interesting parallels to centromeric Ty3/*gypsy*-type retrotransposons bearing CHDs known as chromoviruses, which are found in plants and fungi. For instance, the MAGGY retrotransposon from *M. grisea* targets heterochromatin via interaction with a “canonical” or group I CHD (CHD_I) with histone marks such as H3K9me2 and H3K9me3 [24]. The CHD of Skipper-1 elements is similar to that of *D. discoideum* HP1 (Fig. 4) and is a representative of group I CHDs (CHD_I); this is consistent with its centromeric accumulation. Some plant chromoviruses contain group II (CHD_II) domains that diverged from CHD_I domains and usually lack the first and third conserved aromatic amino acid that form the “cage” required to interact with methylated histone tails [24, 54] (see Fig. 4). CHD_II motifs can tether retrotransposons to heterochromatin without interacting with histone marks [24], yet many CHD_II-bearing chromoviruses are not heterochromatin-associated but spread on chromosomes [55]. CHD_II domains are notably similar to chromo shadow domains (CSD), which are required to mediate the homo- and heterodimerization of HP1 proteins, for instance, in *D. discoideum* [38]. Thus, CSDs may represent protein interaction platforms that mediate the integration of CHD_II-bearing chromoviruses into heterochromatin by recognizing specific heterochromatin-associated factors [24]. It is tempting to speculate that the divergence of CHD_II domains from canonical CHDs in Skipper-2 elements enabled the development of a new integration preference away from centromeric DNA into intergenic regions downstream of tRNA genes. Interestingly, the transition from CHD_I to CHD_II domains in plant chromoviruses was estimated to date back 500-400 mya [54], which is approximately the time (~600 mya) when the dictyostelids began to evolve from their last common ancestor [33].

## Conclusions

In the environments of gene-dense genomes, retrotransposons from organisms as divergent as dictyostelid social amoebae and budding yeast reveal convergent evolution leading to the selection of tRNA gene-flanking sequences as potential safe integration sites. In the evolution of dictyostelids, at least six inventions of targeted integration can be discriminated by the choice of distinct integration windows upstream or downstream of tRNA genes by phylogenetically distinctive retrotransposons. In *D. discoideum*, the strong preference of TRE family retrotransposons to integrate near tRNA genes has likely promoted their expansion to almost 4 % of the genome; however, comparing different dictyostelid genomes suggests that *D. discoideum* is an exception to the rule and may have been affected by an unknown evolutionary force that either demanded or coincidentally enabled a burst of retrotransposon amplification in this particular species. In general, it is evident from our analysis that non-mutagenic retrotransposition is not a license to amplify possibly because host cells keep track of their repetitive sequences to maintain genome stability.

## Methods

Annotated genome sequences of *D. discoideum* [16], *D. purpureum* [34], *D. fasciculatum* [33], and *P. pallidum* [33] were accessed at dictyBase (http://dictybase.org/) [56]. A genome sequence of *D. lacteum* was obtained from Genbank (LODT01000000). The genome sequence of *Protostelium fungivorum* will be reported elsewhere (F.H., T.W., G.G., manuscript in preparation).

To identify new retrotransposons in dictyostelid genomes, known retrotransposon sequences from *D. discoideum* [17] were used as queries in TBLASTX searches with a cutoff value of e < 10^-15^. Found sequences were expanded by 3000 bp upstream and downstream and analyzed using Jemboss [57]. Blast searches were performed to construct consensus sequences from DNA alignments of individual retrotransposon copies. Searches for LTR sequences were performed using LTR_FINDER [58] to determine full-length LTR retrotransposons and to identify solo LTR sequences. Flanking sequences of retrotransposon copies were analyzed for the presence of tRNA genes using tRNAscan-SE [59] and ARAGORN [60]. To specifically search for tRNA gene-associated retrotransposons, tRNA genes were identified genome-wide using tRNAscan-SE [59] and ARAGORN [60] and listed with 3000 bp upstream and downstream flanking regions for BLASTN searches using the aforementioned identified retrotransposon sequences as queries. Consensus sequences of full-length retrotransposons have been deposited in Repbase (http://www.girinst.org/repbase/) [61]. The following elements have alternative names in Repbase: Pp_Skipper-1: Gypsy-1_PPP; Pp_DGLT-A.1: Gypsy-3_PPP; Pp_DGLT-A.2: Gypsy-2_PPP; Pp_DGLT-A.3: Gypsy-4_PPP; Df_Skipper-1: Gypsy-2_DFa; Df_Skipper-2: Gypsy-1_DFa.

For phylogenetic analyses of LTR retrotransposons, the core domains of reverse transcriptase (RT), ribonuclease H (RNH), and integrase (IN) were determined by searching the Conserved Domain Database [62]. Alignments were generated using ClustalX [63], and conserved amino acid positions were highlighted using BoxShade (http://www.ch.embnet.org/software/BOX_form.html). Shading is to a 50 % consensus with black boxes indicating invariant amino acids and gray boxes representing similar amino acids. Phylogenetic analyses were conducted using the MEGA7 software package [64]. Phylogenetic trees were analyzed using the Neighbor-Joining [65] or the Maximum Likelihood method [66] as indicated in the figures.

## Additional file

Additional file 1: Figure S1. Alignments of GPY/F motifs in the carboxy-terminal regions of integrase domains in dictyostelid LTR retrotransposons. **Figure S2.** Alignments of RT, RNH, and IN domains of dictyostelid LTR retrotransposons. **Figure S3.** Phylogenetic analysis of dictyostelid LTR elements. **Figure S4.** Alignment of RT domains of dictyostelid non-LTR retrotransposons. **Figure S5.** Phylogenetic analysis of RT domains of dictyostelid non-LTR elements. **Figure S6.** Alignment of dictyostelid non-LTR elements bearing RNH domains. **Table S1.** Comparison of retrotransposon in annotated dictyostelid genomes. **Table S2.** Association of tRNA genes with TREs in the *D. discoideum* genome. (PDF 7243 kb)

## Abbreviations

APE: Apurinic/apyrimidinic endonuclease
CHD: Chromatin organization modifier domain (chromo domain)
CSD: Chromo shadow domain
DGLT-A: *Dictyostelium* gypsy-like transposable element
DIRS: *Dictyostelium* intermediate repeat sequence
GAG: Group-specific antigen
IN: Integrase
ITR: Inverted terminal repeat
LTR: Long terminal repeat
mya: Million years ago
ORF: Open reading frame
PBS: Primer binding site
PPy: Polypyrimidine stretch
RNH: Ribonuclease H
RT: Reverse transcriptase
TRE: tRNA gene-associated retroelement
