# Supplementary material for: Convergent evolution of tRNA gene targeting preferences in compact genomes
Source: Mob DNA. 2016 Aug 31;7(1):17. doi: 10.1186/s13100-016-0073-9 (PMC5006619; doi:10.1186/s13100-016-0073-9)
Supplement: Additional file 1: Figure S1. — Alignments of GPY/F motifs in the carboxy-terminal regions of integrase domains in dictyostelid LTR retrotransposons. Figure S2. Alignments of RT, RNH, and IN domains of dictyostelid LTR retrotransposons. Figure S3. Phylogenetic analysis of dictyostelid LTR elements. Figure S4. Alignment of RT domains of dictyostelid non-LTR retrotransposons. Figure S5. Phylogenetic analysis of RT domains of dictyostelid non-LTR elements. Figure S6. Alignment of dictyostelid non-LTR elements bearing RNH domains. Table S1. Comparison of retrotransposon in annotated dictyostelid genomes. Table S2. Association of tRNA genes with TREs in the D. discoideum genome. (PDF 7243 kb) [file 13100_2016_73_MOESM1_ESM.pdf]

Additional File 1

Convergent evolution of tRNA gene targeting preferences in compact genomes

Thomas Spaller, Eva Kling, Gernot Glöckner, Falk Hillmann, Thomas Winckler

Content

Figure S1	Alignments of GPY/F motifs in dictyostelid LTR retrotransposons.
Figure S2	Alignments of RT, RNH, and IN domains of dictyostelid LTR retrotransposons.
Figure S3	Phylogenetic analysis of dictyostelid LTR elements.
Figure S4	Alignment of RT domains of dictyostelid non-LTR retrotransposons.
Figure S5	Phylogenetic analysis of RT domains of dictyostelid non-LTR elements.
Figure S6	Alignment of dictyostelid non-LTR elements bearing RNH domains.
Figure S7	Phylogenetic analysis of RNH domains of dictyostelid NLTR elements.
Table S1	Comparison of retrotransposon in annotated dictyostelid genomes.
Table S2	Association of tRNA genes with TREs in the *D. discoideum* genome.

Supplemental references

Figure S1: Alignments of GPY/F motifs in the carboxy-terminal regions of integrase domains in dictyostelid LTR retrotransposons.

Alignments were generated with ClustalX [1] and BoxShade [2]. Black boxes indicate invariant amino acids and gray boxes represent similar amino acids. Shading is to a 50% consensus. Ty3 (GenBank M23367) is shown for comparison.The consensus of this motif as suggested by Malik and Eickbush [3] is indicated. Abbreviations: Dd: *Dictyostelium discoideum*; Dp: *D. purpureum*; Dl: *D. lacteum*; Pp: *Polysphondylium pallidum*; Df: *D. fasciculatum*; Pf: *Protostelium fungivorum*; Sc: *Saccharomyces cerevisiae.*

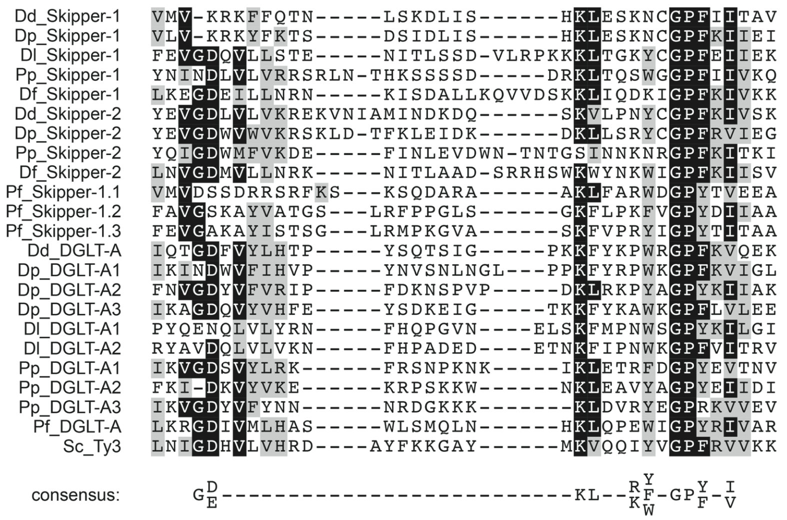

Figure S2: Alignments of RT, RNH, and IN domains of dictyostelid LTR retrotransposons.

Core domains of reverse transcriptase (RT) (**A**), ribonuclease H (RNH) (**B**), and integrase (IN) (**C**) were determined by searching the Conserved Domain Database [4]. Alignments were generated with CLUSTALX [1]. Shading is to a 50% consensus and was generated with BOXSHADE [2]. Black boxes indicate invariant amino acids and gray boxes represent similar amino acids. Active site residues are indicated by red dots. The sequence of Ty3 (GenBank M23367) was aligned for comparison. Abbreviations: Dd: *Dictyostelium discoideum*; Dp: *D. purpureum*; Dl: *D. lacteum*; Pp: *Polysphondylium pallidum*; Df: *D. fasciculatum*; Pf: *Protostelium fungivorum*; Sc: *Saccharomyces cerevisiae.*

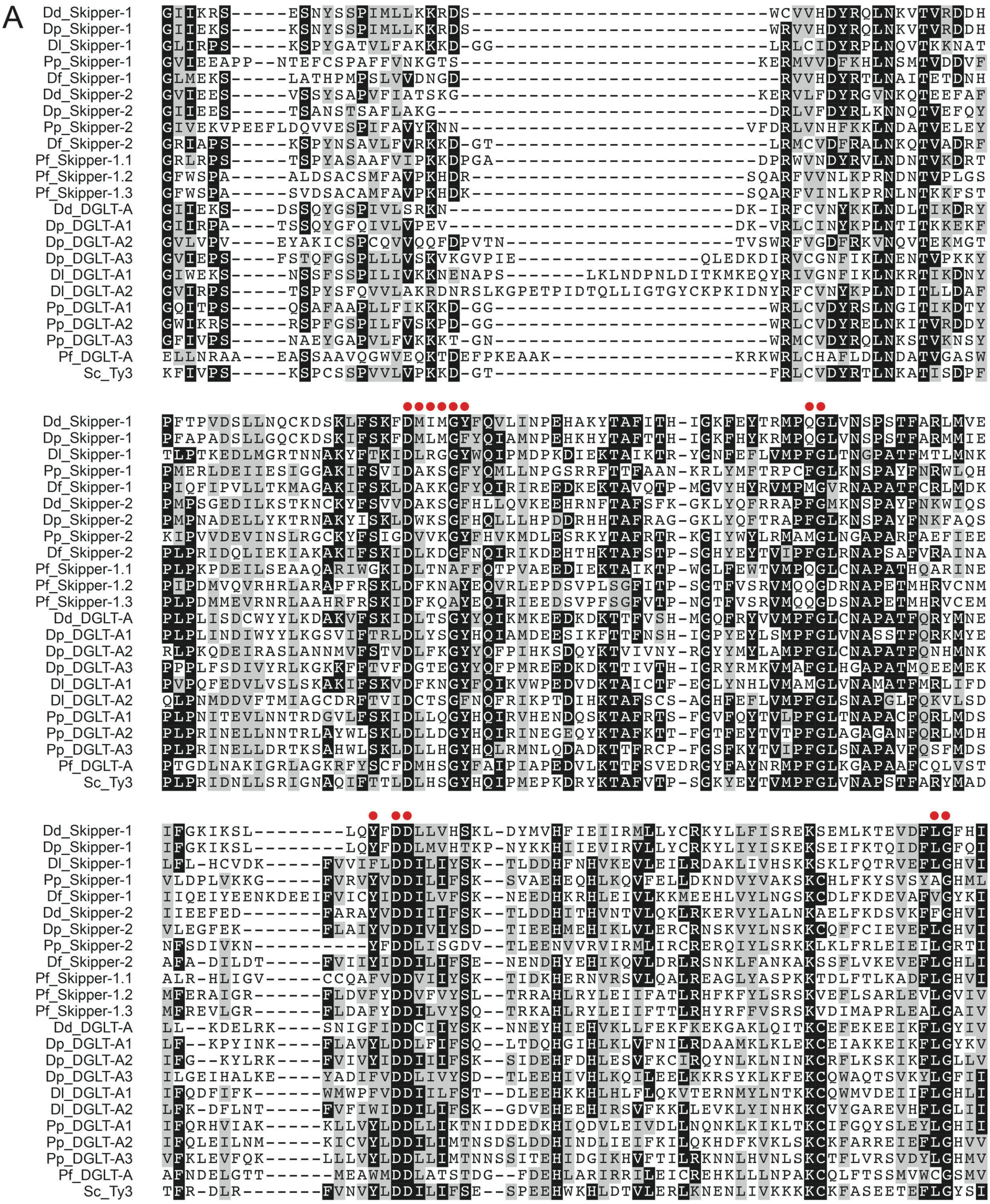

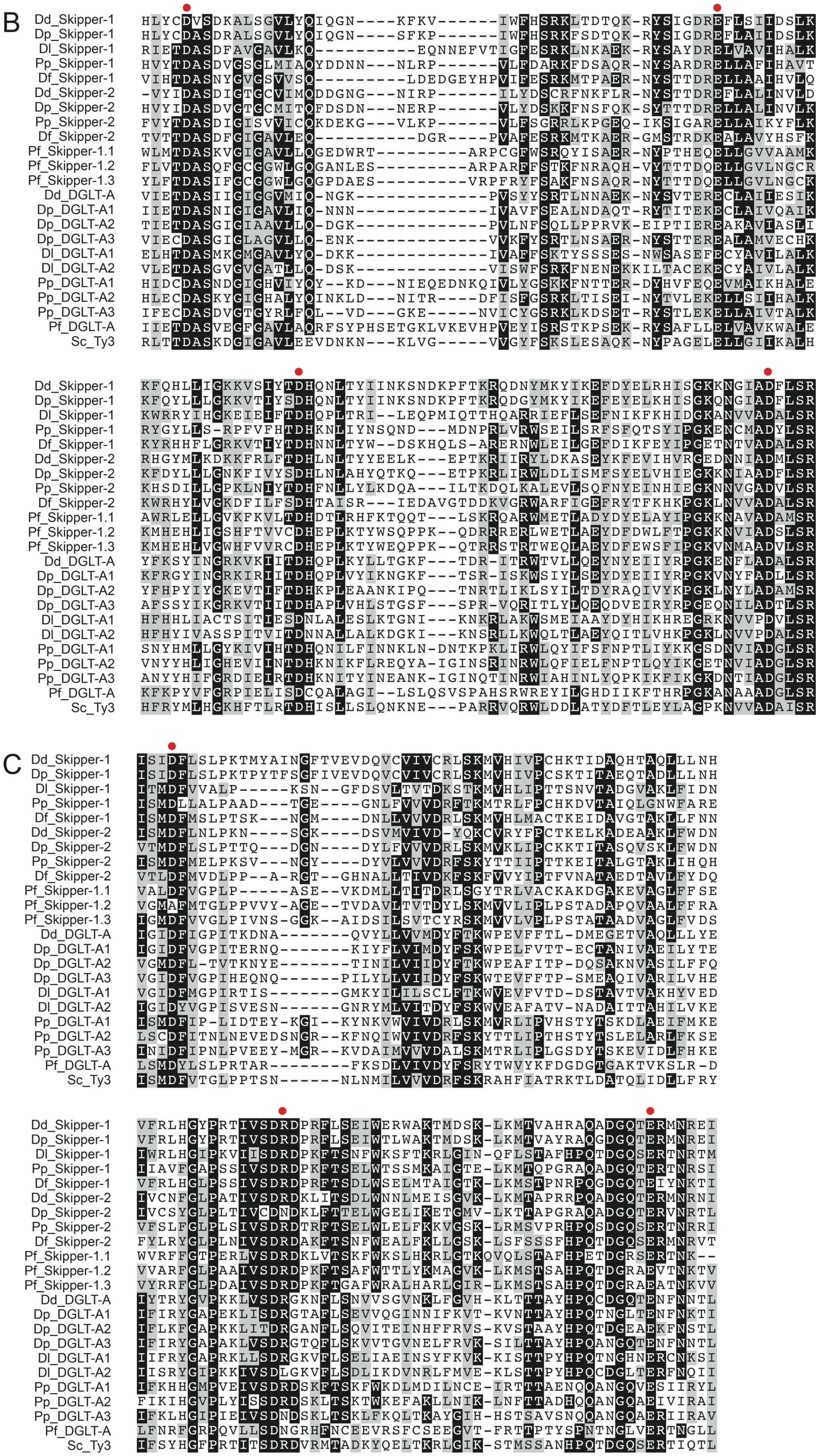
B
Dd_Skipper-1
Dp_Skipper-1
Dl_Skipper-1
Pp_Skipper-1
Df_Skipper-1
Dd_Skipper-2
Dp_Skipper-2
Pp_Skipper-2
Df_Skipper-2
Pf_Skipper-1.1
Pf_Skipper-1.2
Pf_Skipper-1.3
Dd_DGLT-A
Dp_DGLT-A1
Dp_DGLT-A2
Dp_DGLT-A3
Dl_DGLT-A1
Dl_DGLT-A2
Pp_DGLT-A1
Pp_DGLT-A2
Pp_DGLT-A3
Pf_DGLT-A
Sc_Ty3
C

Figure S3: Phylogenetic analysis of dictyostelid LTR elements.

Alignment of the concatenated core domains of RT, RNH, and IN was generated with CLUSTALX and analyzed using the Neighbor Joining method. Numbers next to each node indicate bootstrap values as percentages out of 1000 replicates. All positions containing gaps and missing data were eliminated. The tree is drawn to scale, with branch lengths in the same units as those of the evolutionary distances used to infer the phylogenetic tree. Retrotransposons integrating upstream and downstream of tRNA genes are indicated in red and blue boxes, respectively. Dd: *D. discoideum*; Dp: *D. purpureum*; Dl: *D. lacteum*; Df: *D. fasciculatum*; Pp: *P. pallidum*; Pf: *Protostelium fungivorum*.

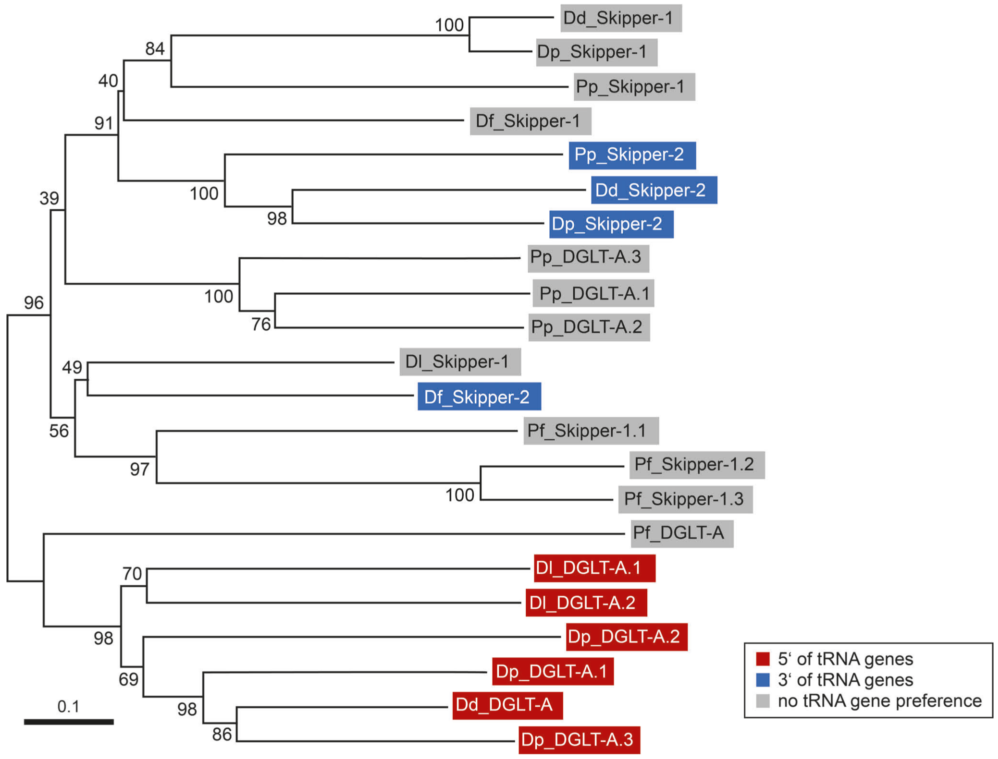

Figure S4: Alignment of RT domains of dictyostelid non-LTR retrotransposons.

Core domains of reverse transcriptases (RT) were determined by searching the Conserved Domain Database [4]. Alignments were generated with CLUSTALX [1]. Shading is to a 50% consensus and was generated with BOXSHADE [2]. Black boxes indicate invariant amino acids and gray boxes represent similar amino acids. Active site residues are indicated by red dots. The sequence of the human L1 element (Hs_L1; U93574) was aligned for comparison. Dd: *Dictyostelium discoideum*; Dl: *D. lacteum*; Dp: *D. purpureum*; Pp: *Polysphondylium pallidum*; Df: *D. fasciculatum.*

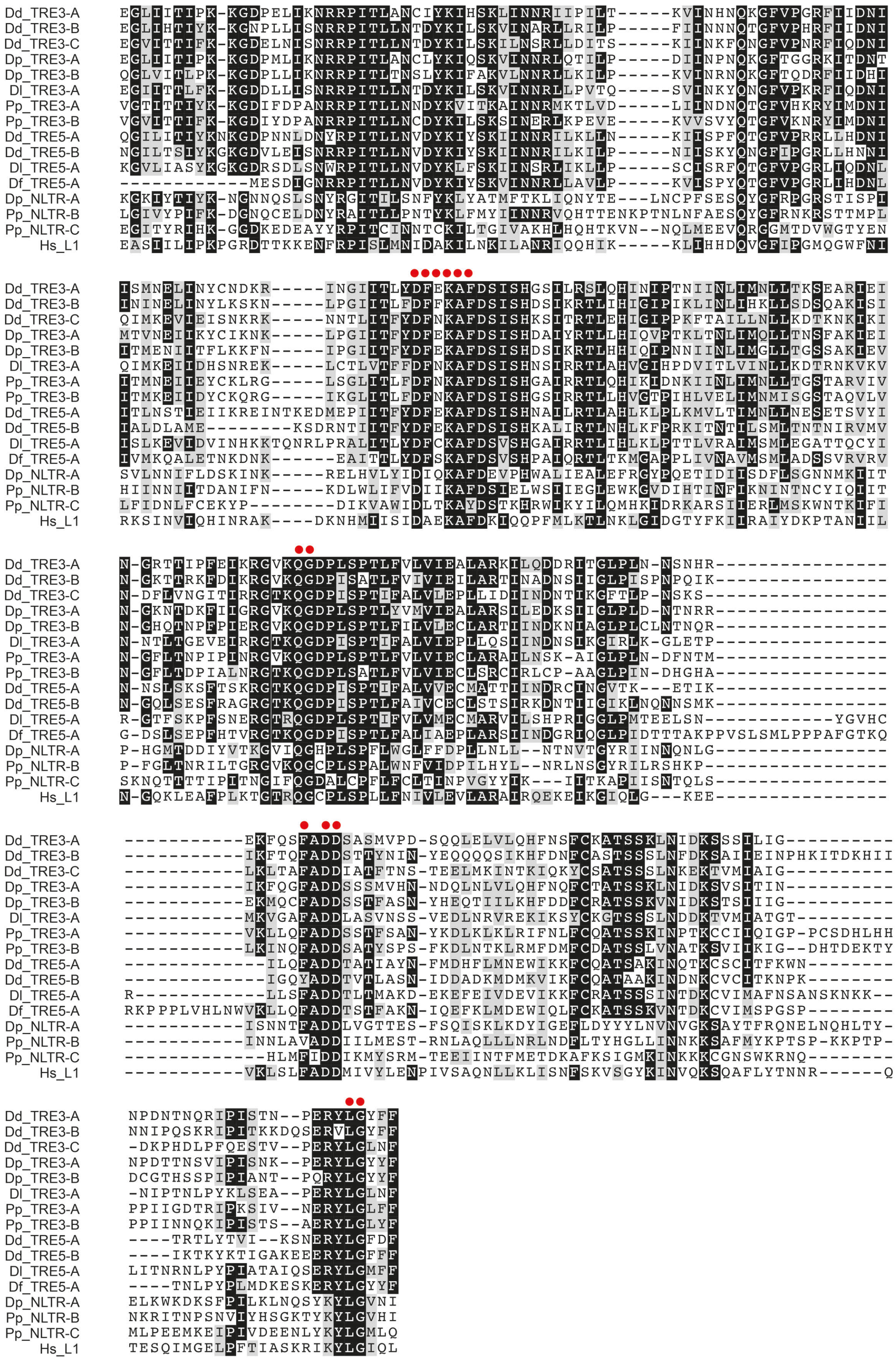

Figure S5: Phylogenetic analysis of RT domains of dictyostelid non-LTR elements.

Alignment of the RT domains was generated and analyzed with MEGA7 [5] using the Neighbor Joining method. All positions containing gaps and missing data were eliminated. Numbers next to each node indicate bootstrap values as percentages out of 1000 replicates [6]. The tree is drawn to scale, with branch lengths measured in the number of substitutions per site. The tree was rooted on RT domains of group II introns.

Sequences used for alignment with dictyostelid NLTR elements were chosen according to a previous phylogenetic analysis performed by Malik et al. [7]: *Trypanosoma brucei* SLACS (X17078), *Trypanosoma cruzi* CZAR (M62862), *Crithidia fasciculata* CRE1 (M33009), *Crithidia fasciculata* CRE2 (U19151), *Drosophila melanogaster* R2 (X51967), *Drosophila mercatorum* R2 (AF015685), *Forficula auricularia* R2 (AF015819), *Bombyx mori* R2 (M16558), *Porcellio scaber* R2 (AF015818), *Bombyx mori* Dong (L08889), *Ascaris lumbricoides* R4 (U29445), *Rattus norvegicus* L1 (U83119), *Mus musculus* L1 (AF081114), *Canis lupus* L1 (AB012223), *Homo sapiens* L1 (U93574), *Oryzias latipes* swimmer (AF055640), *Cyprinodon macularius* swimmer (AF055643), *Xenopus laevis* Tx1 (M26915), *Zea mays* Cin4 (Y00086), *Arabidopsis thaliana* Ta11 (AAC13599), *Dictyostelium discoideum* TRE5-A (X57034), *Chlorella vulgaris* Zepp (AB008896), *Aedes aegypti* JAM1 (Z86117), *Caenorhabditis elegans* RTE1 (AF025462), *Colletotrichum gloeosporioides* Cgt1-3 (L76169), *Magnaporthe grisea* Mgr583 (AF018033), *Ascobolus immersus* Mars1 (X99080), *Neurospora crassa* Tad1 (L25662), *Drosophila melanogaster* R1 (X51968), *Bradysia coprophila* R1 (L00945, *Bombyx mori* R1 (M19755), *Bombyx mori* TRAS1 (D38414), *Aphonopelma sp.* R1 (AF015489), *Bombyx mori* SART1 (D85594), *Anopheles gambiae* RT1 (M93690), *Anopheles gambiae* RT2 (M93691), *Drosophila silvestris* LOA (X60177), *Aedes aegypti* Lian (U87543), *Drosophila miranda* TRIM (X59239), *Drosophila subobscura* Bilbo (U73800), *Drosophila melanogaster* Jockey (M22874), *Drosophila funebris* Jockey (M38437), *Drosophila melanogaster* TART (U14101), *Drosophila melanogaster* Doc (X17551), *Drosophila melanogaster* F (M17214), *Peridroma saucia* YAKPs1, *Drosophila melanogaster* BS (X77571), *Drosophila yakuba* Helena (AF012049), *Drosophila mauritiana* Helena (AF012043), *Bombyx mori* AMY (U07847), *Aedes aegypti* JuanA (M95171), *Culex pipiens* JuanC (M91082), *Chironomus tentans* NCR1Cth (L79944), *Anopheles gambiae* T1 (M93689), *Anopheles gambiae* Q (U03849), *Gallus gallus* CR1 (U88211), *Acanthochelys spixii* CR1 (AB005891), *Schistosoma mansoni* SR1 (U66331), *Biomphalaria glabrata* BGR (X60372), *Drosophila melanogaster* I (M14954), *Drosophila teissieri* I (M28878), *Trypanosoma brucei* ingi (X05710), *Trypanosoma cruzi* L1Tc (X83098). Group II introns: *Streptococcus agalactiae* (WP_000561483), *Leptospira santarosai* (ZP_13300096), *Escherichia coli* (ZP_13884144), *Acetonema longum* (ZP_08623276), *Mycobacterium sp.* (WP_011779439), *Bacillus coagulans* (WP_014095942), *Clostridium kluyveri* (WP_011988638), *Rhodococcus jostii* (WP_011598972), *Frankia sp.* (WP_011436613).

Figure S5 (continued)

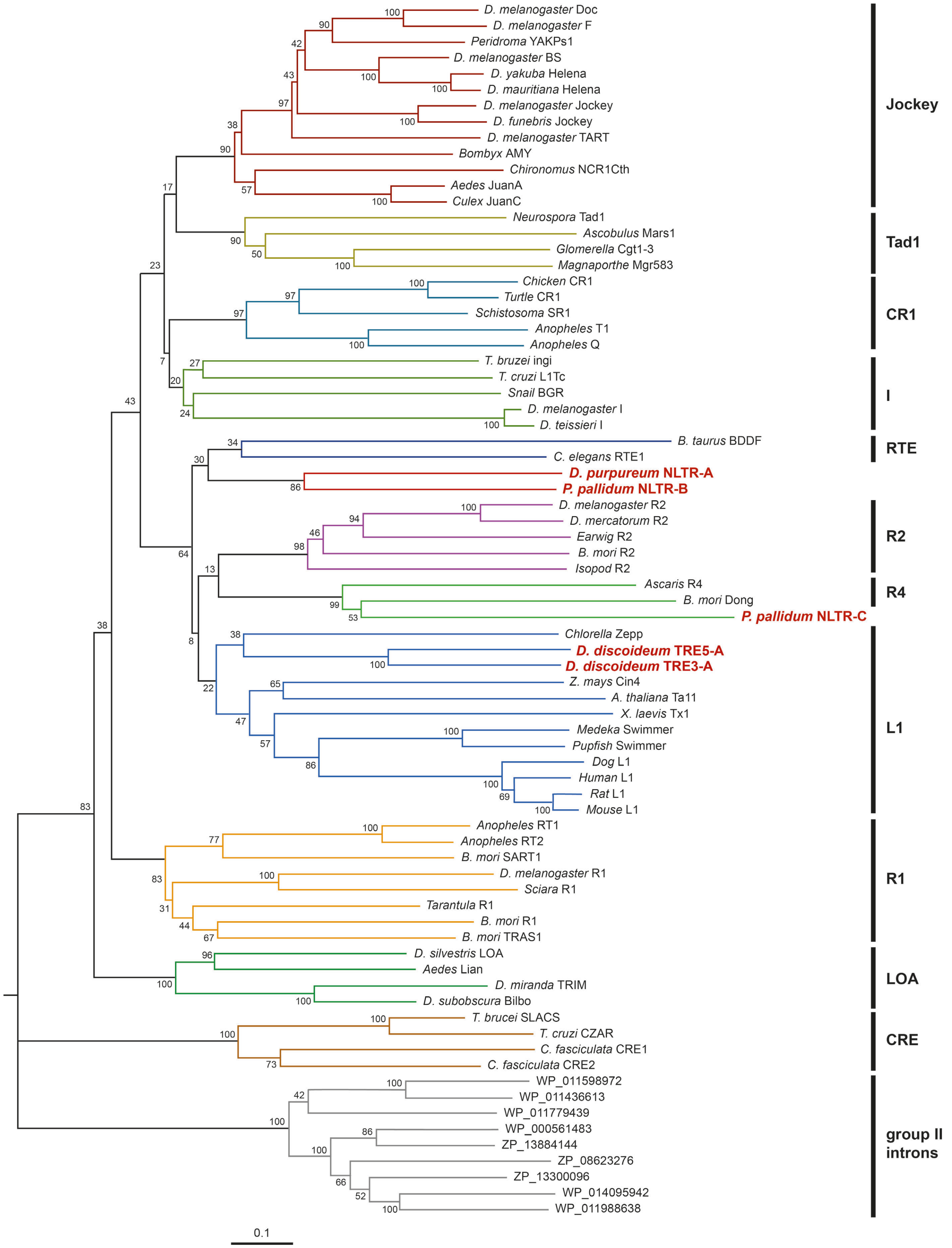

Figure S6: Alignment of dictyostelid non-LTR elements bearing RNH domains.

Core domains of ribonuclease H (RNH) were determined by searching the Conserved Domain Database [4]. Alignments were generated with ClustalX [1]. Shading is to a 50% consensus and was generated with BoxShade [2]. Black boxes indicate invariant amino acids and gray boxes represent similar amino acids. Sequences used for alignment with *D. purpureum* NLTR-A and *P. pallidum* NLTR-B were chosen according to a previous phylogenetic analysis performed by Malik et al. [7]: *Bombyx mori* TRAS (GenBank D38414), *Aphonopelma sp.* R1 (AF015489), *Drosophila melanogaster* I (M14954), *Trypanosoma bruzei* ingi (X05710), *Colletotrichum gloeosporioides* Cgt1-3 (L76169), *Magnaporthe grisea* Mgr583 (AF018033), *Aedes aegypti* Lian (U87543), and *Drosophila miranda* TRIM (X59239).

```
TRAS       RIFTDGSKIEGR------------------------VGAALSIWDGEVEIRSLKLALAPYCTV
R1         QIYTDGSKSESG------------------------TGAAFVVFRNGELWMSRSYKMTASNTS
I          FIFTDGSKINYT--------------------------ISFAITTETDVLKY--GILPPYSSV
ingi       ELWTDGSVSLGE-----------------------KSGAAALLYRNNKLICAPKTGAGELSCS
Cgt1-3     IVYSDGSKAPNG-----------------------ATGFGFVIYRGSRRIAQGCGRLG-IAEV
Mgr583     VVYTDGSEKHENNCV--------------------QIGYGWAAFRAGLEFAARAASITPESHV
Lian       VCYTDGSLLEGR--------------------------AGAGVYSRELRLNQ-FYSLGRNCTV
TRIM       HFYTDGSKLDGR--------------------------VGGGVYCSELEISH-CFRLPDHCSV
Dp_NLTR-A  EFYTDGSYKKINN----------------------KRKAGYGIYNQSENISLN-FIEGTAKTI
Pp_NLTR-B  KIYTDGSCHKQIEKENTTVINNNEILTSTHNIIIRPAKAAFGVYSPSSGYWFN-NRVSGIQGI

TRAS       YQAELLALSYAVKEAQ------LRNG--STFGVFSDSKAALLTVIN--HGSLHPLAVDIRKML
R1         SQAEILAIWKALQWLL------ADGVGIKSCAVITDSQSSLQALAN--PSCDWLLVMRAKAAY
I          LTSETIAILEAIELTK------NRRG---KFIICSDSLSAVDSIQN--TNNNSFYPSRIRSLI
ingi       YRAECVALEIGLQRLLKWL--PAYRSTPSRLSIFSDSLSMLTALQTGPLAVTNPILRRLWRLL
Cgt1-3     FDGEAEGARAGLRRAL------LTSQ-GQPIHICIDNTSVIQGIRS--NIPDS--SQAAFLEI
Mgr583     FDAEAIGALKGLQAAA------KAQP-GARIWICVDSTSVIWGLRG--DAPRS--SQWAFLEF
Lian       FQAEIFALMCGVQSAL------QQRVMGKVIYFCSDSQAAIKALAS--ANSRSKLVIACRTQI
TRIM       FQAEIEAIKEAISIVS------KLRLDTHLVCVFSDSQAAIKALGS--ISSNSATVKDCRRSL
Dp_NLTR-A  TGAELSAPTHILSSYPIN----HKININTDSQVSIDIIESLIQNPYPIQYFGTPFFEIIKKLF
Pp_NLTR-B  ENAELQAILHALQHAPPNTNIEIITDSESSINFITKSTTNKFHLYNHFNKSYFPVLSAITKLI

TRAS       KQCALQNKT----VALYWIKAHAGLEGNERADQLAKEAALLSK
R1         RQLLRNGVA----VRFFWTKGHATCEGNKIADSAAREASASGL
I          TQHAPKIK-------IMWIPGHSGIKGNELADQAAKSASSMPL
ingi       LQVQRRKIR----IRLQFVFGHCGVKRNEVCDEDAKRAADLPQ
Cgt1-3     QAVARIYN-----IQTHWSPGHQGIKGNEEADILAKEGTTLPV
Mgr583     HNLVDLLRKQSTEVRVRWCPGHQGIPGNDRADELAKAGSAGPP
Lian       EELNSVNS-----VNLVWVPGHSSIAGNELADELARDGASHDF
TRIM       HEIAEQLD-----LFLIWVPGHRDIEGNDAADELARQGTTIPL
Dp_NLTR-A  KYKLKRDNFDEKNTNFIKVKAHSNIIGNEIADKLAKKALTLPI
Pp_NLTR-B  NQRKQLNLH----TKLTKVAAHSGNPGNDIADRLAKGAISQPP
```

Table S1: Comparison of retrotransposon in annotated dictyostelid genomes.

	Dictyostelium discoideum	***Dictyostelium purpureum***	***Dictyostelium lacteum***	***Polysphondy-lium pallidum***	***Dictyostelium fasciculatum***
Haploid genome size (Mb)	34	33	23	33	31
Number of genes	12,646	12,410	10,225	12,675	12,430
Gene density (CDS/Mb)	372	376	444	384	401
Number of tRNA genes	418	353	57	273	198
Retrotransposons total (%)	8.1	0.9	0.000003	0.7	0.6
non-LTR retro-transposons (%)	3.7	0.5	0.000002	0.3	0.3
LTR retrotrans-posons (%)	1.1	0.4	0.000001	0.3	0.3
YR retrotrans-posons (%)	3.3	0	0	0	0,1
tRNA gene-targeting retro-transposons (%)	3.8	0.9	0.000002	0.4	0.4
References	[8, 9]	[10]	Genbank LODT01000000	[11]	[11]

CDS: coding sequence; YR: tyrosine recombinase.

Table S2: Association of tRNA genes with TREs in the *D. discoideum* genome.

The individual tRNA gene loci were inspected for the presence of TRE5 or TRE3 elements. The tRNA genes on chromosomes 1-6 were extracted from genome assembly file "*D. discoideum* Non-coding sequences" as of 03-29-2016 (www.dictybase.org). * Note that TRE elements can target the same tRNA genes sequentially, producing tandems. Only the first TRE of such tandems directly adjacent to the targeted tRNA gene was considered in this analysis.

Chromosome	tRNA genes	TREs*	% tRNA genes occupied*
1	79	52	65.8
2	91	56	61.5
3	79	45	57.0
4	33	21	63.6
5	34	18	52.9
6	87	54	62.1
total	403	246	61.0

Supplemental references

1. Thompson JD, Gibson TJ, Plewniak F, Jeanmougin F, Higgins DG. The ClustalX windows interface: flexible strategies for multiple sequence alignment aided by quality analysis tools. Nuc Acids Res. 1997;24:4876-82.
2. BoxShade. http://www.ch.embnet.org/software/BOX_form.html.
3. Malik HS, Eickbush TH. Modular evolution of the integrase domain in the Ty3/gypsy class of LTR retrotransposons. J Virol. 1999;73:5186-90.
4. Marchler-Bauer A, Derbyshire MK, Gonzales NR, Lu S, Chitsaz F, Geer LY et al. CDD: NCBI's conserved domain database. Nucleic Acids Res. 2015;43(Database issue):D222-D6.
5. Kumar S, Stecher G, Tamura K. MEGA7: Molecular Evolutionary Genetics Analysis version 7.0 for bigger datasets. Mol Biol Evol. 2016;Mar 22. pii: msw054. [Epub ahead of print].
6. Felsenstein J. Confidence limits on phylogenies: An approach using the bootstrap. Evolution. 1985;39:783-91.
7. Malik HS, Burke WD, Eickbush TH. The age and evolution of non-LTR retrotransposable elements. Mol Biol Evol. 1999;16:793-805.
8. Glöckner G, Szafranski K, Winckler T, Dingermann T, Quail M, Cox E et al. The complex repeats of *Dictyostelium discoideum*. Genome Res. 2001;11:585-94.
9. Eichinger L, Pachebat JA, Glöckner G, Rajandream M-A, Sucgang R, Berriman M et al. The genome of the social amoeba *Dictyostelium discoideum*. Nature. 2005;435:43-57.
10. Sucgang R, Kuo A, Tian X, Salerno W, Parikh A, Feasley CL et al. Comparative genomics of the social amoebae *Dictyostelium discoideum* and *Dictyostelium purpureum*. Genome Biol. 2011;12:R20.
11. Heidel AJ, Lawal HM, Felder M, Schilde C, Helps NR, Tunggal B et al. Phylogeny-wide analysis of social amoeba genomes highlights ancient originis for complex intercellular communication. Genome Res. 2011;21:1882-91.
